# Supplementary material for: Stress Knowledge Map: A knowledge graph resource for systems biology analysis of plant stress responses
Source: Plant Commun. 2024 Apr 15;5(6):100920. doi: 10.1016/j.xplc.2024.100920 (PMC11211517; doi:10.1016/j.xplc.2024.100920)
Supplement: Document S1. Supplemental Figures 1–4 [file mmc1.pdf]

Plant Communications, Volume *5*

Supplemental information

Stress Knowledge Map: A knowledge graph resource for systems biology analysis of plant stress responses

Carissa Bleker, Živa Ramšak, Andras Bittner, Vid Podpečan, Maja Zagorščak, Bernhard Wurzinger, Špela Baebler, Marko Petek, Maja Križnik, Annelotte van Dieren, Juliane Gruber, Leila Afjehi-Sadat, Wolfram Weckwerth, Anže Županič, Markus Teige, Ute C. Vothknecht, and Kristina Gruden

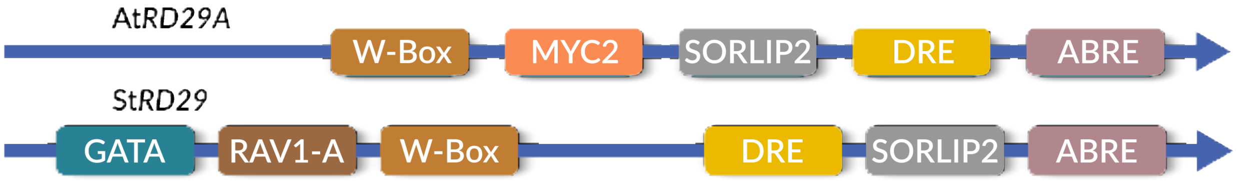

Supplementary Figure 1: Visualisation of abiotic stress related cis-regulatory binding motifs within the 1 kbp upstream region of the transcription initiation site of AtRD29A and StRD29.
ABRE: ABA-Responsive Element; DRE: Dehydration Responsive Elementi; GATA-Box: light responsive GATA motif; SORLIP2: Sequence Over-Represented in Light-Induced Promoters; RAV1-A: RAV1 binding sequence; WRKY: W-Box recognition element; MYC2: basic-helix-loop-helix transcription factor MYC2 binding site.

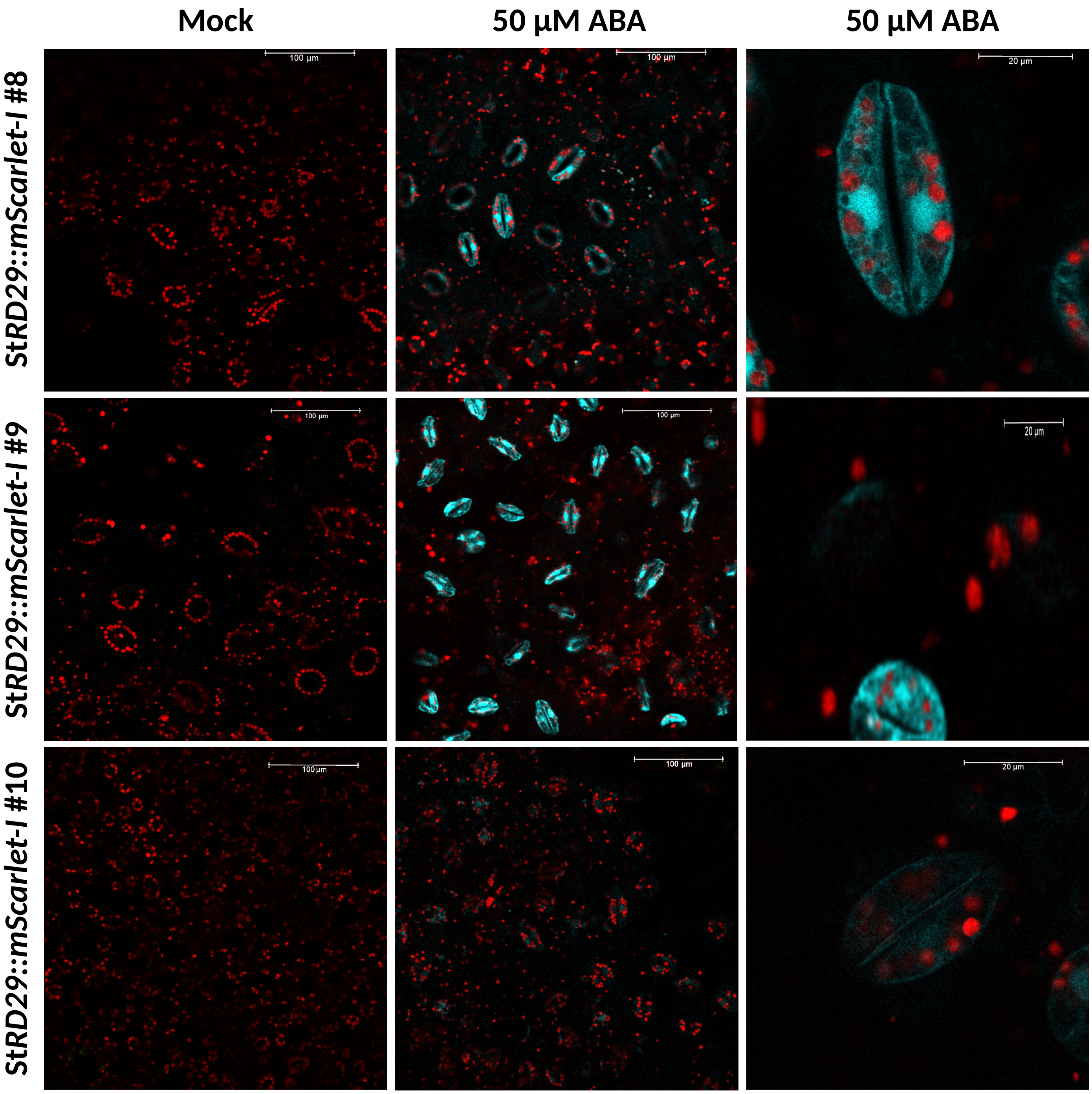

Supplementary Figure 2: Microscopic analyses of the ABA response of *StRD29::mScarlet-I.*
The fluorophore mScarlet-I was expressed under the control of the StRD29 promoter in transgenic potato plants. Leaf discs (7 mm) of three differentlines (#10 with very weak response) were treated with 50 µM ABA (or imaging buffer as a mock control) for 24 hours. After the incubation, mScarlet-I fluorescence was visualized with an excitation at 569 nm and emission was recorded at 585-595 nm (cyan) using a Leica SP8 lightning. Chlorophyll fluorescence (red) was visualized with an excitation at 569 nm with emission recorded at 650 – 705 nm.

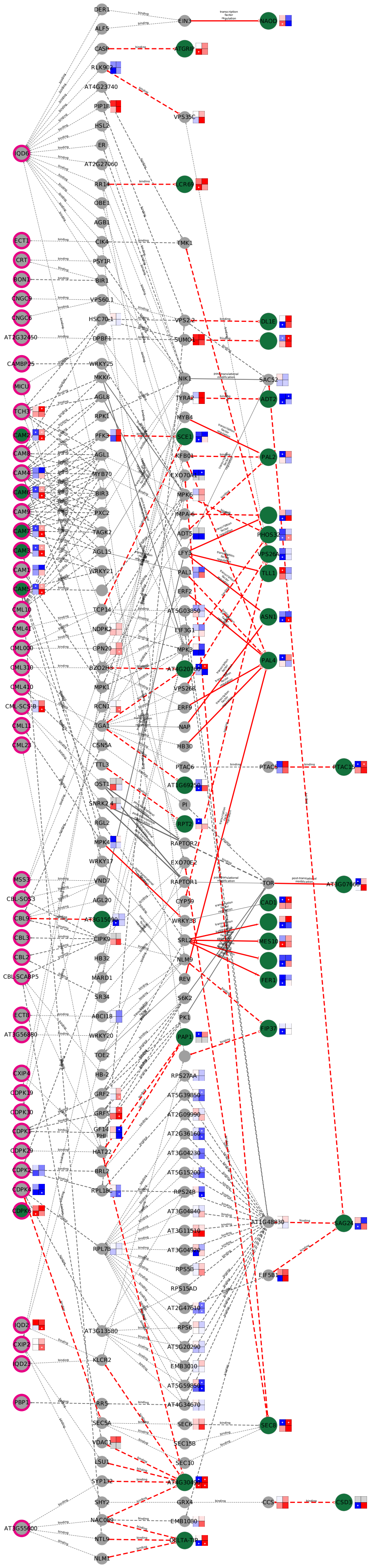

Supplementary Figure 3: Deciphering the Ca^{2+} dependent network in peroxide signalling. All shortest paths identified in CKN leading from known Ca^{2+} related proteins (sources - pink bordered nodes) to Ca^{2+}-dependent redox-responsive proteins identified by proteomics (targets - green filled nodes) using rank 0, rank 1, and rank 2 edges (as described in Table 1 legend), merged into a single network. Legend as in Figure 4.

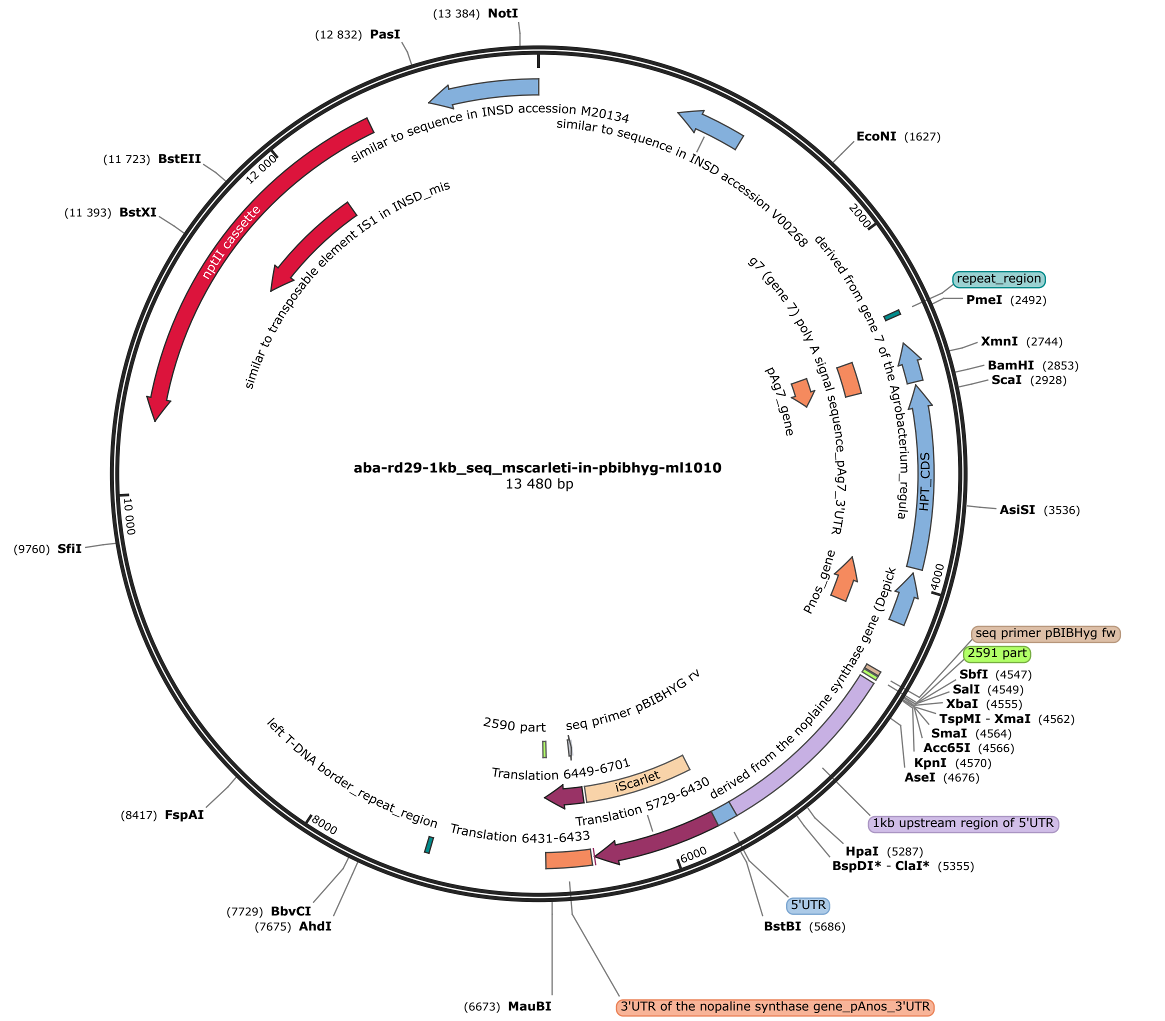

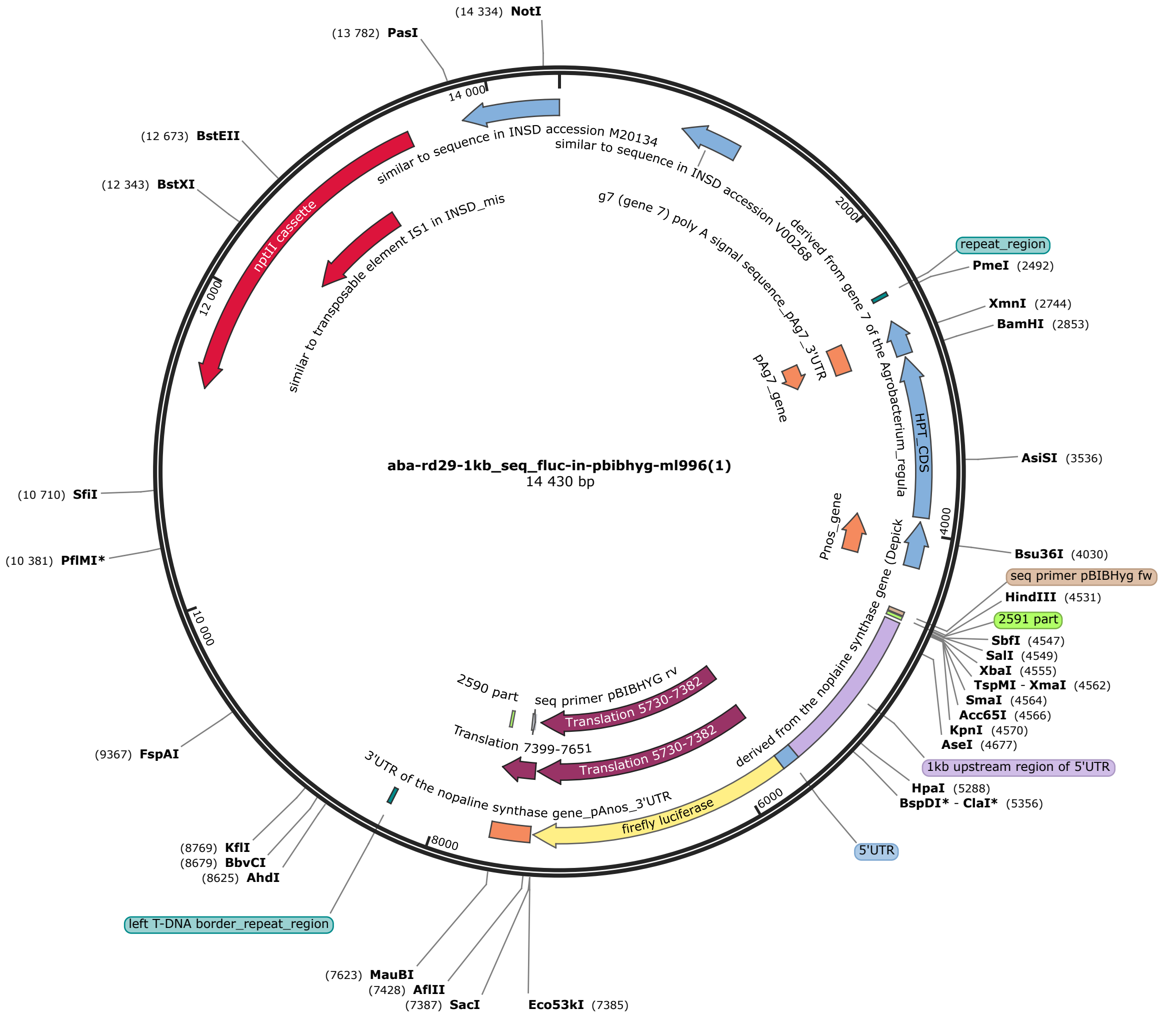

Supplementary Figure 4: Visualisation of the features of Vector pBibHyg carrying StRD29::mScarletI. (above) and StRD29::fluc (below).
