# Supplementary material for: Stress Knowledge Map: A knowledge graph resource for systems biology analysis of plant stress responses
Source: Plant Commun. 2024 Apr 15;5(6):100920. doi: 10.1016/j.xplc.2024.100920 (PMC11211517; doi:10.1016/j.xplc.2024.100920)
Supplement: Document S2. Article plus supplemental information [file mmc12.pdf]

Plant Communications
Resource article

CellPress Partner Journal

Stress Knowledge Map: A knowledge graph resource for systems biology analysis of plant stress responses

Carissa Bleker[1,6,*], Živa Ramšak[1,6], Andras Bittner[2], Vid Podpečan[3], Maja Zagorščak[1], Bernhard Wurzinger[4], Špela Baebler[1], Marko Petek[1], Maja Križnik[1], Annelotte van Dieren[2], Juliane Gruber[4], Leila Afjehi-Sadat[5], Wolfram Weckwerth[4], Anže Županič[1], Markus Teige[4], Ute C. Vothknecht[2] and Kristina Gruden[1,*]

[1]Department of Biotechnology and Systems Biology, National Institute of Biology, Večna pot 121, 1000 Ljubljana, Slovenia

[2]Plant Cell Biology, Institute of Cellular and Molecular Botany, University of Bonn, Kirschallee 1, 53115 Bonn, Germany

[3]Department of Knowledge Technologies, Jožef Stefan Institute, Jamova cesta 39, 1000 Ljubljana, Slovenia

[4]Department of Functional & Evolutionary Ecology, University of Vienna, Djerassiplatz 1, 1030 Vienna, Austria

[5]Mass Spectrometry Unit, Core Facility Shared Services, University of Vienna, Djerassiplatz 1, 1030 Vienna, Austria

[6]These authors contributed equally to this article.

***Correspondence: Carissa Bleker (carissa.bleker@nib.si), Kristina Gruden (kristina.gruden@nib.si)**

https://doi.org/10.1016/j.xplc.2024.100920

ABSTRACT

Stress Knowledge Map (SKM; https://skm.nib.si) is a publicly available resource containing two complementary knowledge graphs that describe the current knowledge of biochemical, signaling, and regulatory molecular interactions in plants: a highly curated model of plant stress signaling (PSS; 543 reactions) and a large comprehensive knowledge network (488 390 interactions). Both were constructed by domain experts through systematic curation of diverse literature and database resources. SKM provides a single entry point for investigations of plant stress response and related growth trade-offs, as well as interactive explorations of current knowledge. PSS is also formulated as a qualitative and quantitative model for systems biology and thus represents a starting point for a plant digital twin. Here, we describe the features of SKM and show, through two case studies, how it can be used for complex analyses, including systematic hypothesis generation and design of validation experiments, or to gain new insights into experimental observations in plant biology.

Key words: knowledge graph, plant stress responses, plant signaling, systems biology, plant digital twin

Bleker C., Ramšak Ž., Bittner A., Podpečan V., Zagorščak M., Wurzinger B., Baebler Š., Petek M., Križnik M., van Dieren A., Gruber J., Afjehi-Sadat L., Weckwerth W., Županič A., Teige M., Vothknecht U.C., and Gruden K. (2024). Stress Knowledge Map: A knowledge graph resource for systems biology analysis of plant stress responses. Plant Comm. **5**, 100920.

INTRODUCTION

The already apparent effects of climate change on agriculture (Shukla et al., 2022), the spread of pests into new regions (Garrett, 2013; IPPC Secretariat, 2021), and rapid population growth (United Nations Department of Economic and Social Affairs Population Division, 2022) present immediate challenges to global food security (Steinwand and Ronald, 2020). Projections show that an increase of up to 75% in crop production is required to meet the 2050 demand (Hunter et al., 2017). This can be achieved with yield improvements through development of stress-resilient crops, a process that requires a holistic understanding of the effects of stressors on plants. The rapid development of modern "omics" technologies enables the generation of large and complex datasets characterizing system-wide responses. To understand the biological meaning of these large-scale datasets and generate meaningful hypotheses, contextualization within current knowledge is needed. We have assembled an integrated resource for plant signaling, Stress Knowledge Map (SKM; https://skm.nib.si), which provides a single, up-to-date entry point for plant-response investigations.

Published by the Plant Communications Shanghai Editorial Office in association with Cell Press, an imprint of Elsevier Inc., on behalf of CSPB and CEMPS, CAS.

This is an open access article under the CC BY license (http://creativecommons.org/licenses/by/4.0/).

SKM integrates knowledge on plant molecular interactions and stress-specific responses from a wide diversity of sources, combining recent discoveries from journal articles with knowledge present in established resources such as KEGG (Kanehisa et al., 2016), STRING (Szklarczyk et al., 2023), MetaCyc (Caspi et al., 2016), and AraCyc (Mueller et al., 2003). SKM extends other aggregated resources (listed in Supplemental Table 1), including the heterogeneous knowledge graphs of KnetMiner (Hassani-Pak et al., 2021), Biomine Explorer (Podpečan et al., 2019), and ConsensusPathDB (Herwig et al., 2016), in that it enables conversion of biochemical knowledge to diverse mathematical modeling formalisms and integration with multi-omics experiments, in addition to enabling interactive exploration of current knowledge that is constantly reproducibly updated. SKM is a versatile resource that assists diverse users, from plant researchers to crop breeders, in investigating current knowledge and contextualizing new datasets in existing plant research. A number of tools have been developed within the SKM environment to support this aim and enable efficient linking to complementary tools.

RESULTS

SKM is a resource that combines two knowledge graphs resulting from the integration of dispersed published information on current biochemical knowledge: the Plant Stress Signaling model (PSS) and the Comprehensive Knowledge Network (CKN) of plant molecular interactions. SKM enables interactive exploration of its contents and represents a basis for diverse systems biology modeling approaches, from network analysis to dynamical modeling.

The Plant Stress Signaling model

PSS is an ongoing endeavor to assemble an accurate and detailed mechanistic model of plant stress signaling by extracting validated molecular interactions from published resources (Miljkovic et al., 2012; Ramšak et al., 2018). Currently, PSS covers the complete stress response cascade within the plant cell (Figure 1), initiating with abiotic (heat, drought, and waterlogging) and biotic stressors (extracellular pathogens, intracellular pathogens, and necrotrophs; Layer 1). Perception of these stressors through diverse receptors (Layer 2) initiates Ca^{2+}, reactive oxygen species (ROS), and MAPK signaling cascades, as well as phytohormone biosynthesis and signaling pathways (abscisic acid [ABA], jasmonic acid [JA], salicylic acid [SA], ethylene, auxin, gibberellins, and cytokinins; Layer 3). These translate perception into a cellular response, resulting in activation of processes that execute protection against stress (Layer 4). Within and across these layers, relevant transcriptional (transcription factors known to act downstream of phytohormones) and posttranscriptional (e.g., small-RNA-transcript regulation known to participate in stress signaling) regulation is included. To capture the relationships between stress responses and growth and development, PSS also contains the major known regulators of growth (target of rapamycin signaling and the above-mentioned hormones) and major primary metabolism processes. Finally, tuberization signaling from potato is included as an example for evaluating potential effects on crop yields.

PSS is based primarily on the model plant *Arabidopsis thaliana* and also contains pertinent information from several crop species, predominantly potato (*Solanum tuberosum*). It currently includes 1425 entities and 543 reactions, a substantial update from the preceding model with 212 entities and 112 reactions (Ramšak et al., 2018). PSS entities include genes and gene products (proteins, transcripts, small RNAs), complexes, metabolites, and triggers of plant stress. Genetic redundancy (Cusack et al., 2021) is incorporated using the concept of functional clusters—groups of genes (possibly across species) that are known to mediate the same functions. Functional clusters can be used to obtain a list of candidate genes linked to a particular use case. For further analysis, individual genes within the functional clusters can be prioritized on the basis of context-specific experimental data (e.g., results of transcriptomics or proteomics analysis). Interactions between these entities include protein–DNA (e.g., transcriptional regulation), non-coding RNA–transcript, and protein–protein interactions, as well as enzymatic catalysis and transport reactions. The majority of these interactions were compiled from peer-reviewed articles with targeted experimental methodology, giving them a high degree of confidence. PSS also contains relevant signaling-associated pathways from KEGG (Kanehisa et al., 2016) and AraCyc (Mueller et al., 2003).

The Comprehensive Knowledge Network

Complementary to PSS, CKN is a large-scale condition-agnostic assembly of current knowledge, offering broader insights into not only stress signaling but also any other plant process. CKN is a network of experimentally observed physical interactions between molecular entities, encompassing protein–DNA interactions, interactions of non-coding RNA with transcripts, posttranslational modifications, and protein–protein interactions (Table 1) in *A. thaliana*. Here we present an update of the previous version, which involved 20 012 entities and 70 091 interactions (Ramšak et al., 2018), to the current version, which provides 30% more entities (26 234 entities) and an almost seven-fold increase in the number of molecular interactions (488 390 unique interactions, Table 1). Entities in CKN include 24 829 of 38 202 genes registered in Araport11 (Cheng et al., 2017).

During the update, only STRING was found to have been altered since 2018 (updated to v.11.5 in 2021) and was thus re-integrated. In addition, nine novel sources of information were added, bringing the total number of sources integrated into CKN to 25 (Supplemental Table 2). Interactions are annotated with the interaction type and whether the interaction has directionality (e.g., undirected binding vs. transcription factor regulation). A ranking system for the interaction reliability (Table 1 legend) enables researchers to evaluate the biological credibility and relevance of individual interactions. CKN includes all relevant reactions from PSS to enable direct comparison of results obtained through both networks.

SKM environment and features

To enable accessibility and exploitation of the resources within SKM, we have developed an encompassing environment (Figure 2). The main features include content exploration and visualization, access to various export formats, and the ability to contribute improvements based on novel biological knowledge. The SKM webpage is publicly available at https://skm.nib.si/.

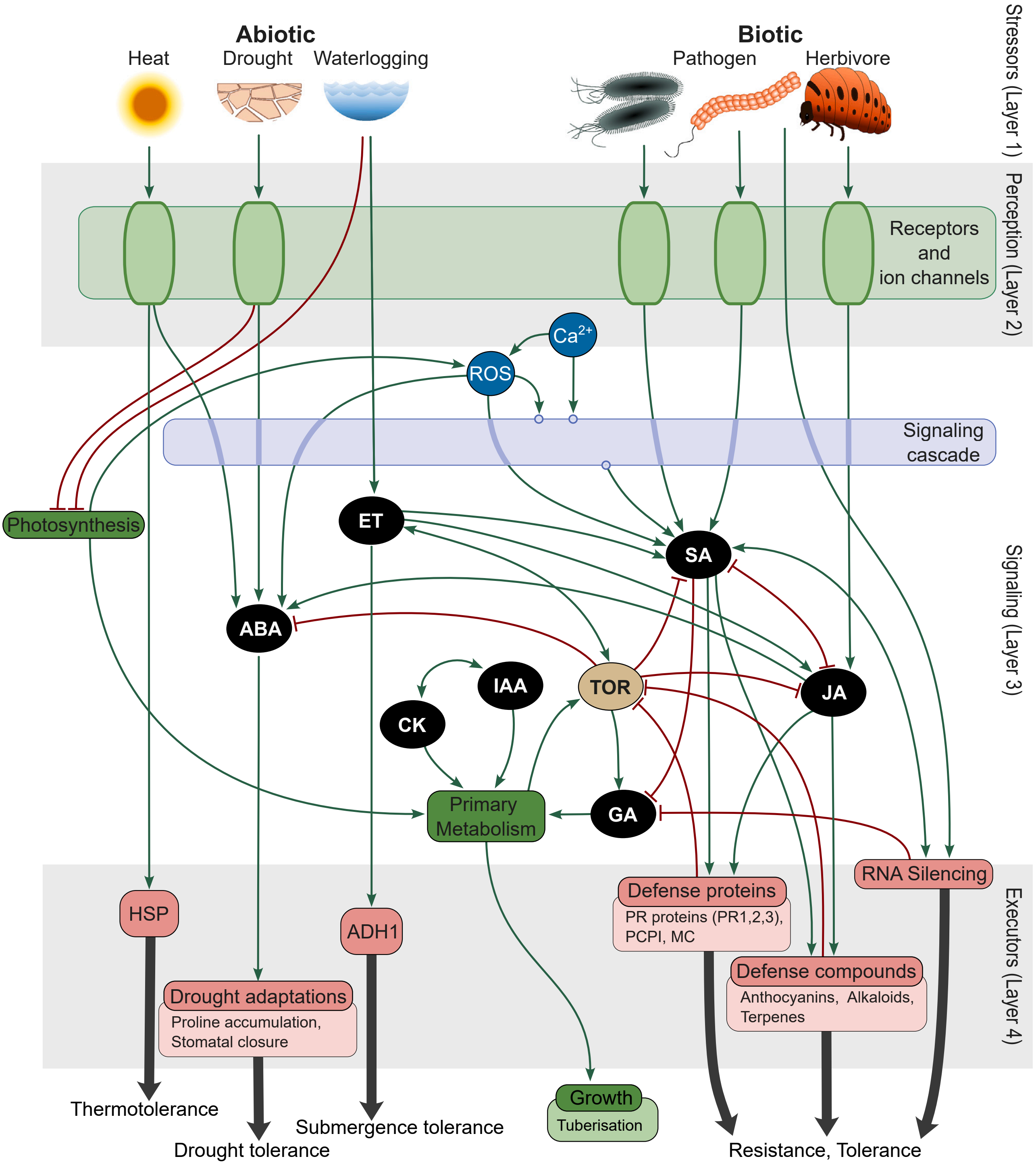

Figure 1. Contents of the Plant Stress Signaling model represented as conceptual layers.

From top to bottom: stressors (Layer 1) acting on the plant are first perceived (Layer 2), resulting in a signaling (Layer 3) cascade that leads to plant defense and/or adaptive changes in the form of executor molecules and processes (Layer 4, examples listed below each group). ABA, abscisic acid; *ADH1*:, alcohol dehydrogenase 1; CK, cytokinin; ET, ethylene; GA, gibberellic acid; *HSP*:, heat shock protein; IAA, indole-3-acetic acid (auxin); JA, jasmonic acid; *MC*:, multi-cystatin; *PCPI*:, potato cysteine proteinase inhibitor; *PR*:, pathogenesis related; ROS, reactive oxygen species; SA, salicylic acid; TOR, target of rapamycin.

Exploration

SKM provides a number of options for the exploration of its contents, including interactive network visualizations of both PSS (PSS Explorer, Figure 2C) and CKN (CKN Explorer, Figure 2F), offering neighborhood extraction of selected entities, shortest-path detection between multiple entities of interest, and on-the-fly exports. Both explorers provide direct references to the object provenance, as well as links for the corresponding *A.*

Interaction type	No. of resources	Rank					Total
		0	1	2	3	4	
Binding	13	650	24 054	30 442	343 401	31 253	429 800
Transcription factor regulation	9	480	1442	8567	174	11 869	22 532
Non-coding RNA interactions[a]	3	–	48	41	34 059	–	34 148
Posttranslational modification	2	754	393	192	–	–	1339
Other[b]	1	571	–	–	–	–	571
Total	25[c]	2455[d]	25 937	39 243	377 634	43 122	488 390

Table 1. Counts of unique CKN interactions by type and reliability ranking.

Rank meanings: 0, manually curated interactions from PSS; 1, literature-curated interactions detected using multiple complementary (mostly targeted) experimental methods (e.g., luciferase reporter assay, co-immunoprecipitation, and enzymatic assays); 2, interactions detected solely using high-throughput technologies (e.g., high-throughput yeast two hybrid assay, chromatin immunoprecipitation sequencing, and degradome sequencing); 3, interactions extracted from the literature (co-citation, excluding text mining) or predicted *in silico* and additionally validated with data; 4, interactions predicted using purely *in silico* binding-prediction algorithms. See Supplemental Table 2 for a detailed list of sources.

[a]Currently only miRNA interactions are included in CKN.

[b]Includes interactions from PSS that do not fall into the previous categories.

[c]Some resources contain multiple interaction types.

[d]Includes interactions expanded from 335 PSS functional clusters to 2253 individual genes.

thaliana genes within the KnetMiner knowledge base (Hassani-Pak et al., 2021), providing even broader context. An additional visualization of the complete PSS model, showing biological pathways, is available in the Newt Viewer (Figure 2D). A separate search interface using internal and external database identifiers (e.g., DOI, KEGG) is also available for PSS.

Modeling and analysis support

PSS is available for download in a number of domain-standard formats (Figure 2H; summarized in Table 2) enabling further visualizations, analysis, and dynamical modeling. A suite of tools implemented in Python (SKM-tools, Figure 2I) has been developed to support additional network analysis of CKN and PSS (described in Table 3).

Extending and improving SKM

The contribution interface of PSS enables constant updates based on novel discoveries (Figure 2B). Registered users can add new entities and interactions to PSS through guided steps, and expert curators are able to make corrections. For major updates to PSS, a batch upload option is also available. The contribution interface automatically retrieves GoMapMan (Ramšak et al., 2014) gene descriptions and short names, as well as article metadata via DOI or PubMed ID, simplifying the contribution process.

FAIRness

SKM has been developed with the FAIR principles (Findable, Accessible, Interoperable, and Reusable) (Wilkinson et al., 2016) at the forefront. SKM is indexed in FAIDARE (FAIR Data-finder for Agronomic Research; https://urgi.versailles.inra.fr/faidare/search?db=SKM), listed in both bio.tools (https://bio.tools/skm) and FAIRsharing.org (https://fairsharing.org/4524), and registered at identifiers.org (https://registry.identifiers.org/registry/skm). Aside from the downloads, a GraphQL endpoint is available for programmatic access to PSS. SKM also makes use of stable reaction and functional cluster identifiers. Data provenance is maintained by storing links to input data through DOIs and external database references (Figure 2G).

Case studies

To showcase the benefits of SKM, we present two case studies demonstrating the use of SKM for contextualization of experimental results within prior knowledge networks. The first case study concerns jasmonates (JA) and SA interference with ABA-mediated activation of *RESPONSIVE TO DESICCATION 29* (*RD29*) transcription, and the second, a proteomics analysis of Ca^{2+}-dependent redox responses.

Case study 1: Interaction of ABA, JA, and SA in the activation of RD29 *transcription*

In *A. thaliana*, the *RESPONSIVE TO DESICCATION 29 A* gene (*AtRD29A*) plays a pivotal role in stress acclimation (Baker et al., 1994) and is transcriptionally regulated via several promoter elements, including the ABA-responsive binding motif ABRE (ACGTG), located close to the transcription initiation site. The 1-kb upstream region of the potato *StRD29* transcription initiation site also contains ABRE and several other abiotic-stress-responsive binding elements (Supplemental Figure 1).

ABA treatment of leaf discs from tobacco plants transiently transformed with *pStRD29::fluc* and from transgenic potato plants (cv. Désirée) carrying the *pStRD29::mScarlet-I* (Supplemental Figure 2) construct strongly induced *pStRD29* activity, which reached its highest amplitude after approximately 4 h in the ABA solution (Figure 3A). Treatments with either jasmonates (JA/MeJA) or SA alone did not lead to an increase in *pStRD29* activity. However, combined treatments of ABA with JA or ABA with SA attenuated the ABA-induced activation of *pStRD29*, revealing a negative effect of both these phytohormones on ABA-dependent *StRD29* transcription (Figure 3A). We subsequently constructed transgenic potato plants (cv. Désirée) carrying the *pStRD29::fluc* construct to confirm the negative effect of MeJA and SA on ABA-responsive promoter activity *in planta* (Figure 3B). The effect of MeJA on ABA activation of both *RD29*s was further analyzed in potato and *A. thaliana* by quantitative real-time PCR. The data revealed attenuation of ABA induction of *RD29A*/*RD29* by jasmonates in both species (Figure 3C).

We first tried to explain the observed effect of jasmonates and SA on ABA-dependent *RD29* activation through promoter motif analysis, but no SA- or JA-signaling-related motifs were identified in

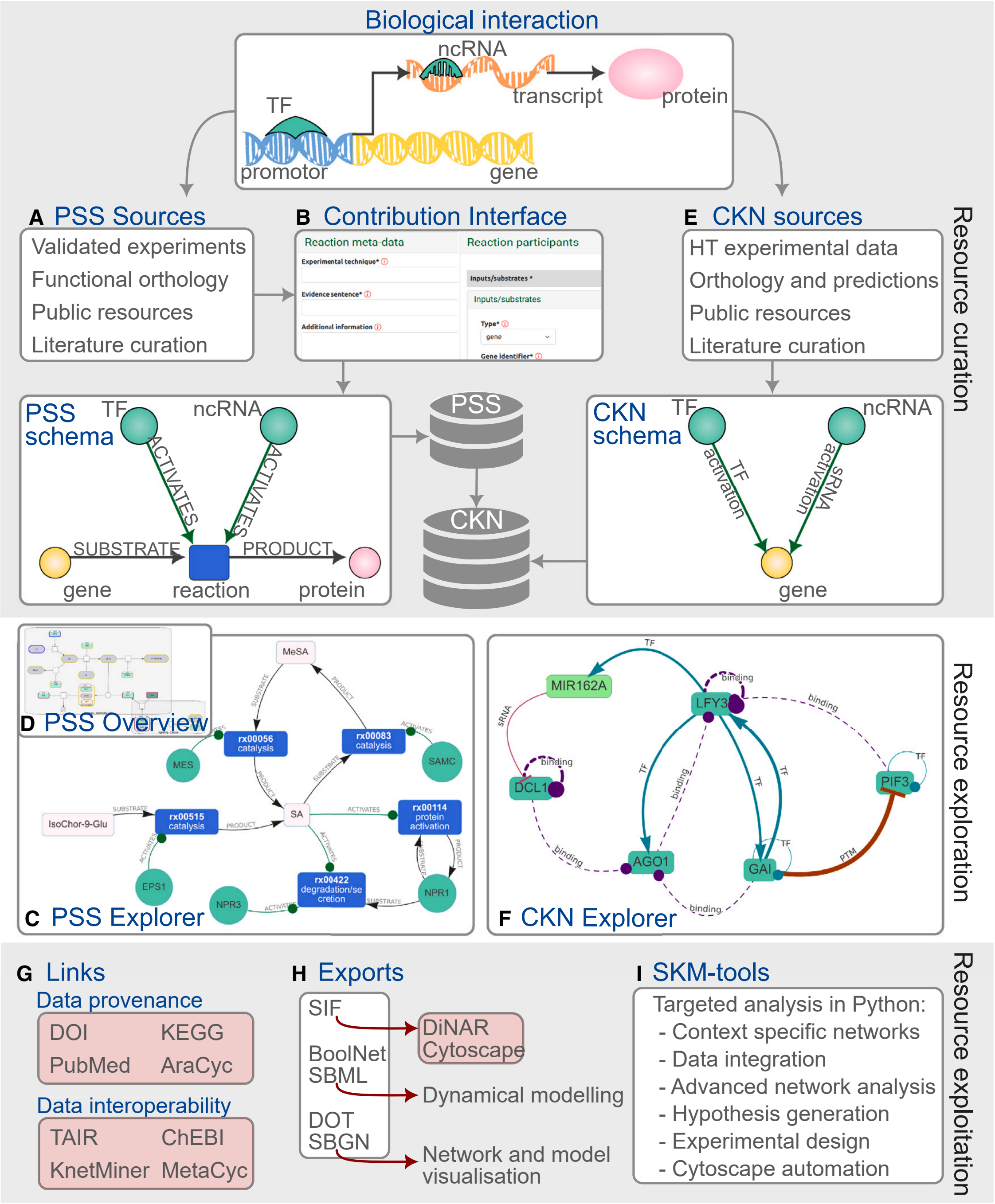

Figure 2. Stress Knowledge Map environment and features.

New validated biological interactions (e.g., transcriptional and translational regulation of a target gene) from various sources **(A)** can be added to PSS through the guided contribution interface **(B)** and are consolidated according to the PSS schema. The contents of PSS can be explored through interactive search and visualization provided by both the PSS Explorer **(C)** and the PSS overview in Newt **(D)**. Correspondingly, sources for CKN interactions **(E)** are integrated and consolidated into the CKN schema through batch scripts and are accessible for exploration through the CKN Explorer **(F)**, which provides interactive search and visualization of CKN interactions. Data provenance and interoperability links **(G)** provide context for SKM contents. Exports of PSS and CKN **(H)** enable various additional analysis and modeling approaches, including through the Python functions provided in the SKM-tools resource **(I)**. Links to specific external resources and tools are highlighted in red. HT, high throughput; PSS, Plant Stress Signaling network; CKN, Comprehensive Knowledge Network; TF, transcription factor; ncRNA, non-coding RNA (currently only miRNAs are included); DOT/SBGN/SBML/SIF, systems biology data formats, see Table 3 for details.

Format	Description	Available for
SBGN-ML	Systems Biology Graphical Notation XML format, enabling graphical visualization of models (Bergmann et al., 2020)	PSS
SBML	Systems Biology Markup Language XML format, enabling mechanistic modeling (Keating et al., 2020)	PSS
DOT	Graph description language compatible with Graphviz applications (Gansner and North, 2000; graphviz.org)	PSS
SIF/LGL	Simple interaction format/large graph format compatible with Cytoscape (Shannon et al., 2003) and DiNAR (Zagorščak et al., 2018)	PSS, CKN
boolnet	Boolean network format for logical modeling compatible with pyboolnet (Klarner et al., 2017) and BoolNet (Müssel et al., 2010), among others	PSS

Table 2. Supported exports of SKM knowledge graphs.

the potato promoter sequence (Supplemental Figure 1). We therefore hypothesized that the signaling pathways interact upstream of actual transcriptional activation. Owing to the complexity of several phytohormone pathway interactions, this was a good case study for the hormone-centric and expert-curated PSS model. We performed a triple shortest-path analysis to identify potential mechanisms of studied crosstalk. This analysis revealed an intersection of JA signaling with the ABA pathway through a protein–protein interaction of the JA-responsive MYC-like transcription factor 2 (MYC2) with the ABA receptor PYRABACTIN RESISTANCE-LIKE 6 (PYL6; Figure 3D). This reaction entry (rx00459) is based on experimental *in vitro* and *in vivo* interaction studies of PYL6 and MYC2 in *A. thaliana* (Aleman et al., 2016). It is conceivable that this interaction depletes PYL, thereby limiting ABA perception (Aleman et al., 2016), which could explain the lower activation of the ABA pathway in the presence of jasmonates. The SA pathway was found to converge with the ABA pathway through the JA pathway with a protein–protein interaction between the SA receptor NPR1 and MYC2 (rx00432) (Nomoto et al., 2021), and this might influence the interaction of MYC with PYL. To verify the hypothesis of direct synergism between JA and SA in attenuation of the ABA response, we performed titration experiments of combined JA and SA treatment on ABA-dependent *StRD29* induction, which was confirmed (Figure 3E; Supplemental Table 3).

Case study 2: Effect of the Ca^{2+} channel inhibitor $LaCl_3$ on proteome-wide peroxide responses

Secondary messengers such as Ca^{2+} and H_2O_2 are important in the translation of many perceived environmental changes towards a cellular response (Kudla et al., 2010; Pirayesh et al., 2021). It is still a challenge to disentangle and understand the principles of specificity and information flow in such networks. Lanthanide ions are known to block anion channels and inhibit the flux of Ca^{2+} across the plasma membrane (Knight et al., 1992; Tracy et al., 2008). Thus, they can be used to identify Ca^{2+}-dependent plant responses. H_2O_2 is known to induce Ca^{2+} transients (Rentel and Knight, 2004). In this case study, we analyzed the proteome of *A. thaliana* rosettes treated with either H_2O_2 or a combination of H_2O_2 and $LaCl_3$ to identify the components of H_2O_2 signaling that are Ca^{2+} dependent. We initially identified 119 proteins whose abundance changed significantly in response to H_2O_2 compared with the mock treatment after 10 or 30 min of treatment. Of these, 49 proteins did not respond significantly in the same manner upon pretreatment with $LaCl_3$ (Supplemental Table 4), indicating that a significant number of H_2O_2-induced changes in protein abundance required a Ca^{2+} signal (Ca^{2+}-dependent redox-responsive proteins).

In the quest to identify mechanistic explanations for these results, CKN provides a universal resource for large-scale hypothesis generation. The largest connected component of CKN contains 98% of the nodes and 99% of the edges, indicating its high connectivity; thus, the analysis was performed on this part of CKN only. Using CKN prefiltered to only leaf-expressed genes, we searched for directed shortest paths from known Ca^{2+}-signaling-related proteins (source set) to the Ca^{2+}-dependent redox-responsive proteins identified by the proteomics approach (target set). The final source set of 53 genes included mainly calmodulins, Ca^{2+}-dependent protein kinases, and calcineurin B-like proteins (Supplemental Table 4). Of the 49 Ca^{2+}-dependent redox-responsive target proteins, 41 were present in CKN. All of these proteins either could be connected to the source set of Ca^{2+}-signaling-related proteins, directly or through an up-to-four-step pathway (Figure 4A), or were in the source set themselves. Combining all the detected shortest paths (all sources to all targets) into a single network (Figure 4A) revealed major network hubs—connected to multiple known Ca^{2+} signaling genes and potentially regulating multiple targets. For example, the analysis revealed an intricate network of calmodulin-dependent regulation of downstream targets in *A. thaliana* (CAM2,3,5,6,7, Figure 4B). Another example of such a hub is Floricaula/leafy-like transcription factor 3 (*LFY3*), shown in Figure 4C, which integrates paths originating from four source nodes and in turn potentially regulates four downstream targets.

The next step in the analysis would be confirmation of the identified mechanisms by functional analysis experiments, e.g., knockout experiments to confirm the role of the proposed regulatory network. The design of such experiments is, however, not always trivial; thus, we designed the CUT-tool within SKM-tools to aid experimentalists. This analysis reveals the minimum interactions that must be severed (“cut-set”) to separate the upstream regulators from the downstream targets. The cut-set to disrupt the regulation of all targets is shown in Figure 4A. As an example, the cut-set of one target, *glutamine-dependent asparagine synthase 1* (*ASN1*), is shown in Figure 4C, revealing that deregulation of *ASN1* would require knockout of both *LFY3* and *A. THALIANA NAC DOMAIN CONTAINING PROTEIN 29* (*NAP*) genes.

Functionality	Description
Load	Directly create networkX (Hagberg et al., 2008) graph objects for PSS or CKN, thus providing access to the multitude of graph analysis and graph operations available in the library
Node filter	For PSS and CKN, filter on node type or node origin (plant or foreign), and additionally for CKN filter nodes based on tissue specificity, creating a network specific to the biological question at hand
Edge filter	Filter CKN edges by rank, removing less reliable edges as the situation requires
Network analysis	Standard node-based analysis approaches, such as neighborhood extraction (identifying the immediate interactors of a node) and shortest-path analysis (identifying directed or undirected paths between source and target nodes of interest)
CUT-tool	CUT-tool provides information on which genes must be perturbed (knockout, knockdown, or overexpression) to modulate the response of the network
Cytoscape automation	Loading of networks and subnetworks into Cytoscape (Otasek et al., 2019); functionalities include providing default styling; node, edge, and path highlighting; network layout from coordinates; and pdf exporters
Multi-omics data visualization	Import of multi-omics experimental data tables (e.g., logFC and *p* values) as context to the networks and functionality to visualize experimental data associated with nodes in the network, through rendering of PNGs (e.g., heatmaps) per node in the Cytoscape view
Link to DiNAR	Instructions for the use of CKN or PSS as the prior knowledge network for integration and visualization of multiple-condition high-throughput data in the DiNAR application (Zagoršča k et al., 2018)

Table 3. Features of SKM-tools.

DISCUSSION

Plant stress signaling pathways are connected by synergistic and antagonistic interactions in a complex network that checks and balances the plant's response to its environment and its growth/development (Eckardt, 2015; Bittner et al., 2022). To understand the functioning of these complex processes, novel approaches are required. Knowledge graphs, such as those provided by SKM, provide powerful and accessible tools to integrate and simplify interpretations within curated published knowledge, as well as providing a basis for a plant digital twin and all the advantages of *in silico* simulation experiments it enables. A number of tools have been developed within the SKM environment to support this and also enable efficient linking to complementary tools.

To showcase the applicability of SKM, we investigated two distinct experimental datasets. In the first, our experiments provided evidence that jasmonate and SA treatment attenuates ABA-activated transcription of *RD29* in both the crop plant potato and the model plant *A. thaliana* through hormonal signaling cross-talk (Figure 3). A manual attempt to extract known information on the crosstalk between ABA and JA with a search in PubMed ((JA OR jasmon*) AND (ABA OR abscisic) AND (plant)) resulted in over 2000 published items. With the wealth of data generated these days, it would be laborious for an individual researcher to perform a thorough literature survey; instead, interrogation of SKM provided a mechanistic hypothesis that explained the experimental results within hours. The hypothesis was empirically supported by further experiments and provides an explanation for the synergistic action of jasmonates and SA that is sometimes argued for in the literature (Mur et al., 2006; Zhang et al., 2020). However, additional experiments are needed to determine (and potentially confirm) whether the exact synergistic mechanism lies in the NPR1–MYC2–PYL6 interaction. Although knowledge compiled in SKM is predominately based on *A. thaliana*, this use case clearly shows its applicability to other species. Through orthology tools such as PLAZA (Van Bel et al., 2022), the knowledge graphs in SKM can be translated to other species, as was done with the previous version of CKN for *Prunus persica* (Foix et al., 2021), *S. tuberosum* (Ramšak et al., 2018), and *Nicotiana benthamiana* (Juteršek et al., 2022). This way, canonical principles of plant signaling networks can be assessed across species.

Our second case study showed that SKM is not only helpful in revealing mechanisms in complex pathways for a single target but also can be used to identify regulators using a large number of targets, as is commonly the case with interpretation of large omics datasets. Using network analyses, arguably the simplest qualitative modeling approach, we identified hubs involved in complex redox–Ca^{2+} signaling interconnectedness. By identifying connections from known Ca^{2+}-related proteins to our experimentally derived target list, we were able to prioritize certain processes and hypotheses in an informed manner. The majority of our targets were found to have only one or two intermediary nodes between them and the upstream Ca^{2+}-related proteins. Paths with many intermediary nodes are less likely to be valid sources of regulation; however, examples of longer paths such as these are known to be functional in the cell, e.g., MAPK signaling cascades. One of the SKM-tools features, the CUT-tool, was designed to help in the next step of research: validation of generated hypotheses. It enables the design of complex functional validation experiments (e.g., gene knockout or overexpression) identifying the genes whose activity should be modulated to achieve a desired effect, taking network redundancy into account.

Overall, in both case studies, SKM proved to be a useful generator of potential mechanistic explanations for the observed data. As with any hypothesis, further validation is needed and some may not prove as valid. More likely hypotheses for further research can be prioritized by weighing the interaction reliability (edge ranks) and exploring the linked content in other resources.

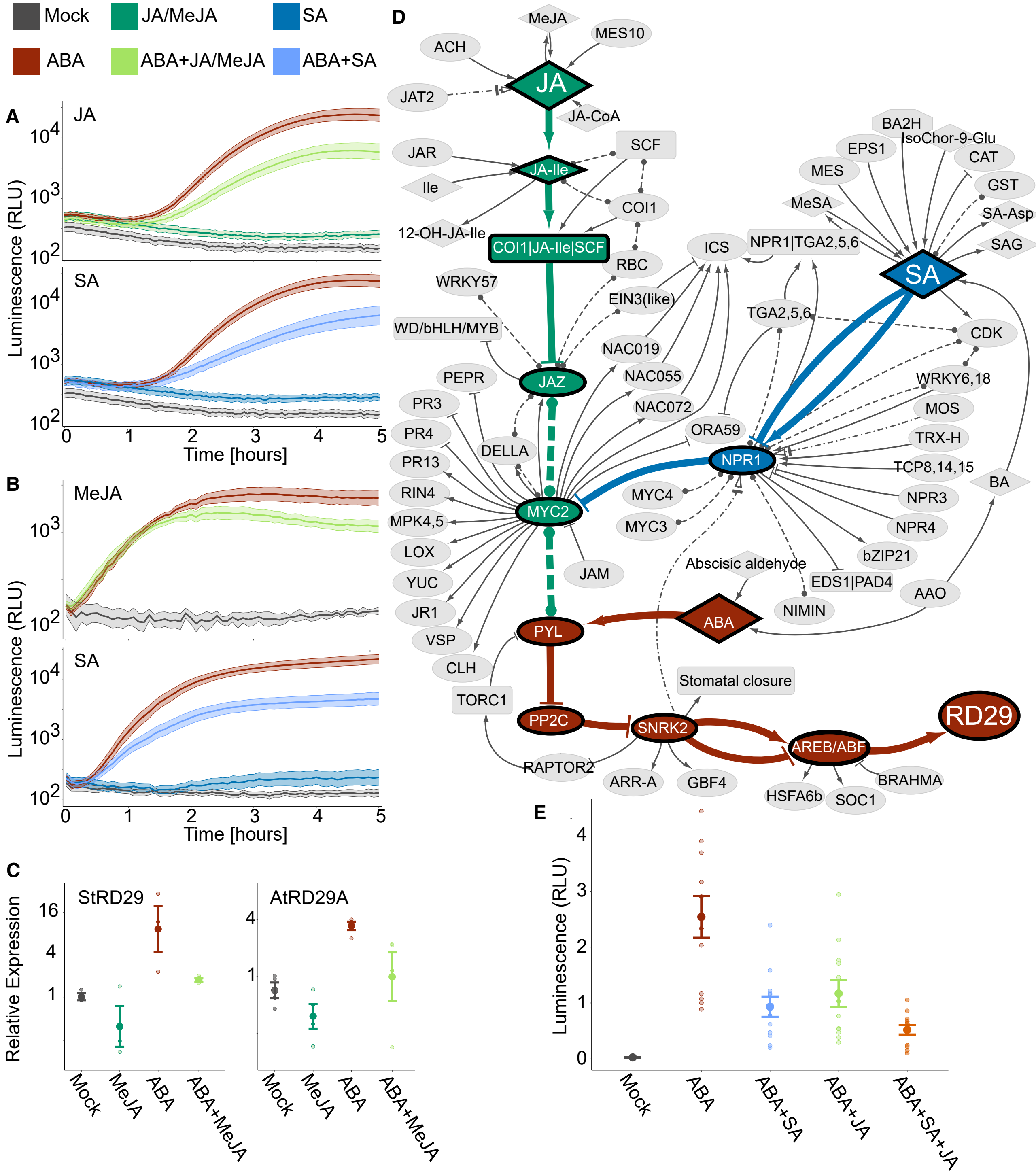

Figure 3. Elucidating connections from JA and SA to ABA-mediated regulation of *RD29* expression in potato.

(A and B) Expression of firefly luciferase driven by the *StRD29* promoter (*pStRD29*::fluc) in **(A)** transiently transformed tobacco leaves treated with the indicated hormones (25 μM JA, 50 μM ABA, and 50 μM SA) and **(B)** transgenic potato leaves treated with the indicated hormones (50 μM MeJA, 50 μM ABA, and 50 μM SA). Values are shown as mean ± SE. Data are provided in Supplemental Table 3.

(C) Relative transcript abundance of *StRD29* (left) and *AtRD29A* (right) 6 h after application of 50 μM ABA, 50 μM MeJA, or a combination of both, analyzed by quantitative real-time PCR. Bars represent mean values ± SE of three or four independent biological replicates.

(D) PSS node-induced subnetwork of shortest paths and immediate neighbors. Paths are directed from the hormones (source) to *RD29* (target). Nodes and edges are colored by the path source: ABA (brown), JA (green), and SA (blue). Edges to first neighbors, edges not on the directed shortest paths, and

(legend continued on next page)

Plant digital twins, virtual replicas of physical systems, are expected to provide a revolutionary platform for modeling the effect of crop management systems and environmental changes in agriculture (Pylianidis et al., 2021). Digital twins can be used to perform *in silico* experiments that guide or replace lab and field experiments. The detail that digital twins provide, combined with fast computational methodologies, enables efficient planning of experiments and will thus speed up our understanding of plant function and provide information for more effective breeding. Aside from being a tool for the interpretation of experimental data, SKM also provides a starting point for the integration of stress signaling and growth trade-offs in digital twins.

SKM will be continuously updated, keeping abreast of the latest developments in the field. Future plans include extending the repertoire of stressors to include additional factors such as cold, salinity, or nutrient deficiencies. We believe the integrated knowledge in SKM will help in understanding plant interactions with the environment by enabling exploration of knowledge and by supporting diverse mechanistic modeling approaches. This is of interest to the wider plant scientific community, enabling the informed design of experiments and, in the long term, contributing to the breeding of improved varieties and precision agriculture.

METHODS

PSS construction

From the predecessor model (PIS v.2; Ramšak et al., 2018), numerous improvements, additions, and reformulations were carried out, resulting in the current PSS. In addition to intracellular pathogens (potyviruses), we extended PSS to also contain perception of extracellular pathogens (*Pseudomonas* sp.) and insect pests, as well as heat, drought, and waterlogging stress. Downstream of perception, PSS now includes Ca^{2+} signaling, ROS signaling, and the MAPK signaling cascade, as well as the synthesis and signaling of all major phytohormones. We also added the synthesis of actuator molecules and processes, as well as known regulators of growth and major processes leading to growth.

PSS is implemented as a Neo4j graph database. The types of nodes and edges (relationships) in the database are summarized in Supplemental Table 5. Genes and gene products are represented by functional cluster nodes, including protein and non-coding RNA nodes. Functional clusters enable the representation of genetic redundancy. These groups were defined using sequence similarity among genes (orthologs and paralogs) and experimental data that confirmed functional overlap. The functional cluster concept includes groupings of enzyme-coding genes (similar to the EC number system), as well as genes involved in transcriptional and translational regulation. Users can access the same information in PSS at the gene level by utilizing the gene-level representation of PSS interactions in CKN. Groups of metabolites with the same biological function are also represented as metabolite families. Nodes also include more abstract entities, such as known but unidentified gene products and plant processes. Finally, foreign entities, such as biotic or abiotic stressors, are also included as nodes.

In addition to biological entities, molecular interactions are also represented by nodes in PSS and are categorized into 10 formal reaction types (e.g., protein activation or catalysis, Supplemental Table 5). Reaction participant nodes are connected to the reaction nodes by relationships, with the type of relationship representing the role of the participant (e.g., SUBSTRATE, ACTIVATES), as demonstrated in Figure 2B. These relationships are annotated with the subcellular location and the form of the participant when involved in the reaction (e.g., "cytoplasm" or "nucleus" and "gene" or "protein").

Where applicable, nodes are annotated with their provenance (e.g., a DOI) and additional information such as biological pathways, gene identifiers, descriptions and annotations (TAIR [Berardini et al., 2015] and GoMapMan [Ramšak et al., 2014]), references to external resources (DOI, PubMed, KEGG [Kanehisa et al., 2016], MetaCyc [Caspi et al., 2016], AraCyc [Mueller et al., 2003], and ChEBI [Hastings et al., 2016]), and explanatory statements (such as a quote from the article and the experimental techniques used in the original experiments).

PSS is available in a number of standard systems biology formats, including SBML (using libSBML [Bornstein et al., 2008]), SBGN (using libSBGN [König, 2020] and pySBGN [Podpečan, 2023] libraries), DOT (using pygraphviz [Aric et al., 2024] and pydot [Sebastian et al., 2023]), and a Boolean formulation in boolnet format. SKM also supplies several generalized formats of PSS in JSON and TSV, enabling multiple formulations of the network model.

All updates to PSS are immediately available in the various interfaces and in all download formats (https://skm.nib.si/downloads). A frozen version (PSS v.1.0.0) is also available in all export formats, and a database dump with detailed deployment instructions can be accessed at GitHub (https://github.com/NIB-SI/skm-neo4j). All sources and resources used to create PSS v.1.0.0 are available in Supplemental Table 6.

CKN construction

The second edition of CKN (CKN v.2) was created by merging pairwise interactions from 25 public resources (details in Supplemental Table 2). Additional filtering was performed on the STRING v.11.5 network (Szklarczyk et al., 2023), where the requirement was to only include physical interactions confirmed by experimental data or existence in a database. As Table 2 summarizes, five reliability ranks were designed to describe the reliability of the interactions across the diversity of the various sources. All interactions were then integrated, resulting in a single network of 574 538 interactions. The network was then condensed by collapsing multiple interactions of the same type between a pair of interactors into a single edge. In this process, the highest ranked interaction took precedence to define the interaction type, but all sources that contained any interaction between the pair were retained in the edge attributes.

All gene loci nodes were annotated using Araport11 (Cheng et al., 2017) downloaded from TAIR in June 2023 (Berardini et al., 2015). Gene loci that had been merged or made obsolete were renamed or removed, respectively. Genes are also annotated with Plant Ontology annotations from TAIR (Berardini et al., 2015) (based on gene expression patterns reported in publications), enabling the extraction of tissue-specific interaction networks.

CKN v.2 is available as part of the SKM application and on the downloads page (https://skm.nib.si/downloads).

shared neighborhood nodes are indicated in gray. Solid edges indicate activation (arrowhead) or inhibition (T head), dashed edges represent binding, and dot–dash edges indicate transport.

(E) Verification of the hypothesis presented in **(D)**. Concentrations of hormones are 50 μM ABA, 15 μM JA, and 30 μM SA. Luciferase activity at 5 h is shown (see Supplemental Table 3 for complete response curves). The results show that SA and jasmonates indeed act synergistically on attenuation of ABA signaling, as the addition of SA and JA has a stronger effect than the addition of each hormone individually.

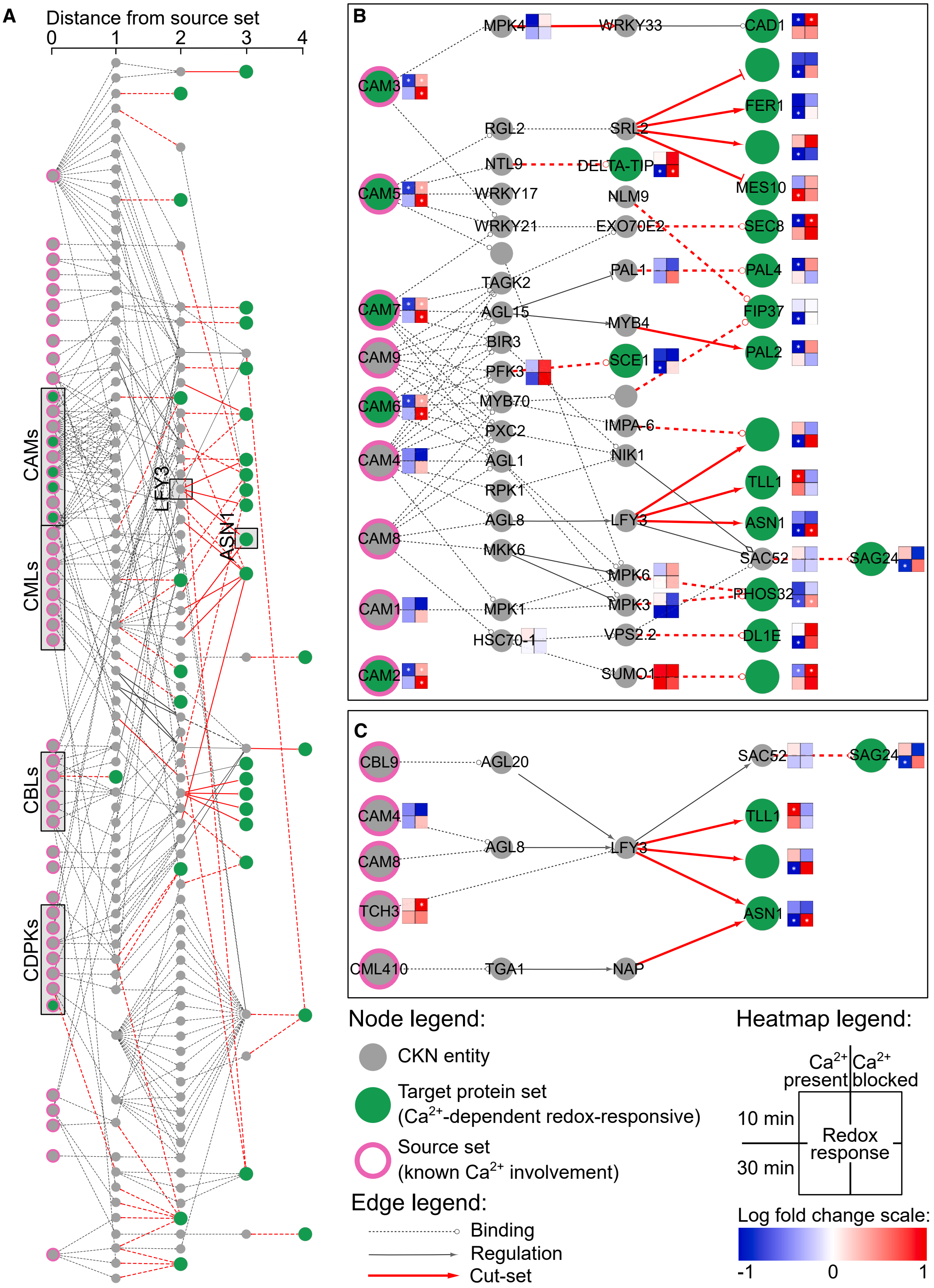

Figure 4. Deciphering the Ca^{2+}-dependent network in peroxide signaling.

(A) All shortest paths identified in CKN leading from known Ca^{2+}-related proteins (sources, pink-bordered nodes) to Ca^{2+}-dependent redox-responsive proteins identified by proteomics (targets, green-filled nodes) using rank 0, rank 1, and rank 2 edges (as described in the Table 1 legend), merged into a single network. The excerpts show **(B)** a subnetwork with a focus on calmodulins and **(C)** a subnetwork with a focus on LFY3 and ASN1. Solid edges with

(legend continued on next page)

SKM environment

The SKM web application is implemented in Python using the microframework Flask. The interactive visualizations of PSS and CKN are based on Biomine Explorer (Podpečan et al., 2019) implemented using vis.js and open-source Python libraries (including networkX [Hagberg et al., 2008] and graph-tools [Peixoto, 2014]) and are freely available on GitHub at https://github.com/NIB-SI/ckn_viz and https://github.com/NIB-SI/pss_viz, respectively. The mechanistic interface to PSS is provided through an instance of the Newt Editor (Balci et al., 2021) using the SBGN standard.

SKM-tools

SKM-tools (https://github.com/NIB-SI/skm-tools) is a collection of Python scripts and notebooks, incorporating network analysis and visualization tools, that facilitates interrogation of CKN and PSS with targeted questions beyond the scope of the web application. Included functionalities are described in Table 3. The tools are developed using the networkX (Hagberg et al., 2008) and py4cytoscape (Ono et al., 2021) libraries.

The CUT-tool makes use of the max-flow min-cut (Edmonds and Karp, 1972) algorithm, which determines the minimum edges that must be severed ("cut-set") to separate the upstream sources from the downstream targets. A max-flow min-cut analysis of multiple sources to an individual target reveals the minimum cut-set needed to disrupt all signaling to the target. To calculate the max-flow min-cut across multiple sources, a dummy node connected with arbitrarily high capacity to all original sources is introduced, and the calculation is performed using the dummy node as the source.

Case studies

Promoter analysis

Predicted *cis*-regulatory motifs within the 1-kb promoter sequences of *AtRD29A* and *StRD29* were identified with the Atcis database of the *A. thaliana* Gene Regulatory Information Server (Lichtenberg et al., 2009). In addition, we used PlantPAN 3.0 (Chow et al., 2019) to identify *StRD29*-specific motifs that were not previously identified in *AtRD29A*.

Plant material and growth conditions

S. tuberosum (cv. Désirée) plants were propagated by cuttings from sterile-grown plants. After 7 days of sterile growth on $^1/_2$ MS medium (pH 5.7, 2% [w/v] sucrose) to initiate root growth, plantlets were transferred to individual pots filled with soil (9 parts soil, 1 part Perligran). *A. thaliana* (ecotype Col-0) seeds were sown directly onto soil and transferred into individual pots after 4–6 days. All experiments used leaves from 18- to 21-day-old plants grown in climate chambers (20°C ± 2°C) under long-day conditions (16 h light/8 h dark) with a light intensity of 120 μmol photons m^{-2} s^{-1} (Philips TLD 18W alternating 830/840 light color temperature).

For promoter reporter assays of transiently transformed *N. benthamiana* leaves, seeds were germinated on Profi substrate (Gramoflor). Five days after germination, seedlings were separated into pots of 15.5 cm diameter × 12 cm height filled with substrate (3 parts Profi substrate, 1 part vermiculite, 1.5 kg Osmocote Start/m^3). Plants were grown in a greenhouse under long-day conditions (16 h light at 28°C/8 h dark at 22°C) with an average light intensity of ~250 μE and 80% relative humidity.

Soltu.DM.03G017570 was identified as the orthologous locus of *A. thaliana RD29A* in *S. tuberosum* cultivar DM1-3 using the DM v.6.1 database (http://spuddb.uga.edu/). To generate the gene reporter lines in the potato cultivar Désirée, 1158 bp of the 5′ UTR directly upstream of the start codon region was amplified by PCR and either the firefly luciferase (fluc) or the *mScarlet-I* (*mScar*) gene in a custom variant of the pBIB Hyg vector carrying hygromycin resistance for selection in plants. The complete sequences of both vectors, including annotations, can be found in Supplemental Figure 4. Both constructs were introduced into the potato cultivar Désirée as described previously (Rocha-Sosa et al., 1989).

Plate-reader-based luciferase assays

Agrobacteria carrying the *pBIN-StRD29::fluc* or *pBIN-AtRD29A::fluc* plasmid were grown in LB liquid medium supplemented with the respective antibiotics. Overnight cultures were diluted to OD600 = 0.1 with fresh LB medium and grown to OD600 = 0.8. Cells were harvested by centrifugation (22°C, 15 min, 4000 *g*) and resuspended in 5% sucrose solution in H_2O to OD600 = 0.2. The agrobacterium suspension was infiltrated into leaves 6, 7, and 8 of 4-week-old *N. benthamiana* plants. Care was taken that the *N. benthamiana* plants selected for infiltration and measurement were not suffering an obvious pathogen attack before infiltration and during the transformation period, hormone treatment, and measurement. After 48 h, leaf discs (ø 6 mm) of infiltrated plants were transferred into 96-well plates containing 100 μl buffered MS (5 mM MES [pH 5.8]) supplemented with 1% sucrose (w/v) and incubated for 2 h under greenhouse growth conditions. Immediately before measurement, luciferin, to a final concentration of 30 μM, and the hormones, to the final concentrations indicated in the text, were added to each respective well. For all combinatorial hormone treatments, the different hormones were applied at the same time to the indicated final concentrations. Fluc luminescence was recorded in a multi-mode microplate reader (TECAN Spark multimode microplate reader, serial no. 2301004717) in a window from 550 to 700 nm for 2 s every 5 min for each well. During the measurement period, the leaf discs were kept in darkness at a constant temperature of 22°C.

For luminescence measurements of *S. tuberosum StRD29::fluc* plants, leaf discs (ø 6 mm) were placed in 96-well plates containing 100 μl of 30 μM luciferin dissolved in $^1/_2$ MS. After 2 h of preincubation, the solution was replaced by 100 μl of 30 μM luciferin containing various effectors (50 μM ABA, 50 μM MeJA, or both). Since MeJA is rapidly hydrolyzed to JA (Stuhlfelder et al., 2002; Wu et al., 2008), JA and MeJA treatments are comparable when eliciting a jasmonate response. Luminescence was measured every 5 min for up to 12 h using a TriStar2 LB 492 multimode reader (Berthold Technologies, Germany). During the measurement period, the leaf discs were kept in darkness. All luminescence analysis was performed with at least five independent experimental replicates. Luminescence data are available in Supplemental Tables 3 and 7.

Transcript analysis

For analysis of *StRD29* and *AtRD29A* transcripts, *S. tuberosum* or *A. thaliana* plants were treated with water (mock), 50 μM ABA, 50 μM MeJA, or a combination of both for 6 h in three or four independent biological replicates. Total RNA was extracted from 100 mg of leaf material using the Gene Matrix Universal RNA Purification Kit (Roboklon, Germany) according to the manufacturer's instructions. RNA integrity was assessed by agarose electrophoresis and RNA quantity and purity with a UV-vis spectrophotometer (Eppendorf, Germany). For quantitative real-time PCR, RNA was transcribed into cDNA using the RevertAid First Strand cDNA Synthesis Kit (Thermo Scientific, Germany). The reaction was stopped by a 5-min incubation at 75°C.

Where applicable, all primers were designed to span exon–intron borders using QUANTPRIME (Arvidsson et al., 2008) (gene identifiers and primer

arrowheads indicate directed, regulatory interactions (see Table 1), whereas dashed edges indicate undirected binding. Red edges are part of the merged cut-set. Nodes with proteomics measurements are annotated with a heatmap indicating the change in protein abundance after 10 min (top row) and after 30 min (bottom row) between H_2O_2- and mock-treated samples (left column) and between Ca^{2+} blocker treatment and H_2O_2 and Ca^{2+} blocker treatment (right column). Significant changes in abundance are marked with a white asterisk in the center of the square. Red, increase in treatment compared with control; blue, decrease in treatment compared with control. Nodes are labeled with their short names, where available. The complete network is provided in Supplemental Figure 3, and all source and target nodes are listed in Supplemental Table 4.

sequences in Supplemental Table 8). Quantitative real-time PCR was performed with three technical replicates for each sample in 96-well plates using a CFX96 real-time thermal cycler system (Bio-Rad, Germany). Each reaction contained 1× SYBR Green master mix (Thermo Fisher), 2 ng/μl cDNA, and the respective forward + reverse primers at 10 μM each. The specificity of each product was assessed on the basis of melting curves after 40 cycles of amplification. All transcript levels were normalized against the geometric mean of the transcript abundances of the reference genes *YLS8* and *CYP5* for *A. thaliana* and *YLS8* and *ACT7* for potato. Target relative copy numbers were calculated using quantGenius (http://quantgenius.nib.si/; Baebler et al., 2017), provided in Supplemental Table 9.

PSS network analysis

We identified the pathway between ABA and *RD29* by querying for all directed shortest paths from ABA to *RD29* in the reaction participant bipartite projection of PSS. We then extracted all directed shortest paths from JA and SA to *RD29* that partially overlapped with the ABA-to-*RD29* path. For added context to these results, we expanded the network induced by the shortest paths to include the first neighbors of all nodes (Figure 3E).

Analysis was performed in Python using the networkX library (Hagberg et al., 2008) and visualized in Cytoscape (Cline et al., 2007) using the py4cytoscape library (Ono et al., 2021). All code is available in the SKM-tools repository (https://github.com/NIB-SI/skm-tools).

Proteomic analysis

Complete rosettes of 3-week-old *A. thaliana* plants were incubated in 1 mM $LaCl_3$ solution or ddH_2O for 1 h. Afterward, plants were transferred into either 20 mM H_2O_2 or ddH_2O and harvested after 10 or 30 min of incubation. Complete rosettes of 12 plants per treatment were pooled and immediately frozen in liquid nitrogen. Frozen plant material was homogenized using a precooled mortar and pestle and stored at −80°C. For peptide isolation, 500 mg of frozen plant material was mixed with 2 ml Lacus buffer (20 mM Tris [pH 7.7], 80 mM NaCl, 0.75 mM EDTA, 1 mM $CaCl_2$, 5 mM $MgCl_2$, 1 mM DTT, 1/200 mM NaF) containing 4 tablets of protease inhibitor (Roche cOmplete, EDTA-free, Protease inhibitor cocktail tablets) and 10 tablets of phosphatase inhibitor (Roche PhosSTOP) per 200 ml. Samples were incubated for 10 min on ice and then centrifuged at 15 000 *g* for 10 min at 4°C. The supernatant was transferred to a new tube, adjusted to 20% (v/v) trichloroacetic acid, and incubated overnight at −20°C. The precipitated samples were stored until preparation for mass spectrometry analysis.

Samples were centrifuged at 15 000 *g*, vacuum-dried, and eluted in urea lysis buffer (8 M urea, 150 mM NaCl, and 40 mM Tris–HCl [pH 8]). Protein concentration was determined via BCA assay (Thermo Fisher). In total, 3 mg of protein per sample was first reduced in 5 mM DTT and then alkylated in 15 mM iodoacetamide for 30 min at room temperature in the dark. The alkylated samples were quenched by adding DTT to a final concentration of 5 mM and mixed with 30 mg Sera-Mag carboxylate-modified magnetic beads (1:1 ratio of hydrophilic and hydrophobic beads, Cytiva, USA). The peptides attached to the beads were washed four times with 80% (v/v) ethanol and digested in a 30 mM ammonium bicarbonate buffer (pH 8.2) containing 30 μg trypsin (Promega, WI, USA). Tryptic digestion was performed overnight at 37°C under constant shaking. The digestion was stopped by the addition of formic acid (end concentration, 4%). In total, 100 μg of digested peptides per sample was transferred to a new reaction tube, vacuum-dried, and stored at −20°C until high-pressure liquid chromatography–tandem mass spectrometry (MS/MS) analysis.

The purified tryptic peptides were dissolved in 0.1% (v/v) formic acid in high-purity water. Approximately 1 μg of peptides was separated by an online reversed-phase high-pressure liquid chromatography apparatus (Thermo Scientific Dionex Ultimate 3000 RSLCnano LC system) connected to a benchtop quadrupole orbitrap (Q-Exactive Plus) mass spectrometer (Thermo Fisher Scientific). The separation was carried out on an Easy-Spray analytical column (PepMap RSLC C18, 2 μm, 100 Å, 75 μm i.d. × 50 cm, Thermo Fisher Scientific) with an integrated emitter, and the column was heated to 55°C. The liquid chromatography (LC) gradient was set to a 140-min gradient method, with a flow rate of 300 nl/min. The LC gradient was set to 5%–50% buffer B (v/v) (79.9% ACN, 0.1% formic acid, 20% ultra-high purity H_2O [MilliQ]) for 125 min and then to 80% buffer B over 5 min.

LC eluent was introduced into the mass spectrometer through an Easy-Spray ion source (Thermo Scientific) with the emitter operated at 1.9 kV. The mass spectra were measured in positive ion mode, applying a top 15 data-dependent acquisition. A full mass spectrum was set to 70 000 resolution at *m/z* 200 (automatic gain control target at 1e6, maximum injection time of 120 ms, and a scan range of 400–1600 [*m/z*]). The mass spectrometry scan was followed by an MS/MS scan at 17 500 resolution at *m/z* 200 (automatic gain control target at 5e4, 1.6 *m/z* isolation window, and maximum injection time of 80 ms). For MS/MS fragmentation, the normalized collision energy for higher-energy collisional dissociation was set to 27%. Dynamic exclusion was set to 40 s, and unassigned and +1, +7, +8, and >+8 charged precursors were excluded. The intensity threshold was set to 6.3e3, and isotopes were excluded. The analysis was performed with five independent experimental replicates for each sample.

Peptide identification and quantification

Identities and peptide features were defined by the peptide search engine Andromeda, which was provided by MaxQuant software (v.2.1.3.0, Max Planck Institute of Biochemistry), using standard settings (Tyanova et al., 2016b). In detail, trypsin-based digestion of the peptides with up to two missing cleavage sites was selected. Methionine oxidation as well as N-terminal acetylation was set as a variable modification for peptide identification. In total, up to three potential modification sites per peptide were accepted. The identified peptide sequences were searched and aligned against the Araport11 (Cheng et al., 2017) reference protein database. The false discovery rate cutoff for protein identification and side identification was set to 0.01. The minimum peptide length was 7 amino acids, and the maximum length was 40 amino acids. For each identified protein group, label-free quantitation intensities were calculated and used for further analysis (Supplemental Table 4).

Potential contaminants and reverse-sequenced peptides were removed before statistical analysis. Only proteins that were detected in at least three of five replicates in at least one treatment group were considered for statistical analysis, which was performed using Perseus (v.2.0.7.0) (Tyanova et al., 2016a). Missing values were replaced by sampling from a normal distribution using the default settings. Protein groups with an absolute fold change of greater than 1.5 compared with the control and a false discovery rate value below 0.05 were considered significantly regulated (Supplemental Table 4).

To filter for Ca^{2+}-regulated proteins, significantly up(down)regulated proteins in La^{3+} + H_2O_2-treated samples compared with La^{3+}-only-treated samples were subtracted from the list of significantly up(down)regulated proteins in H_2O_2-treated samples. An additional filtering step was performed to ensure a compelling difference in abundance between the two contrasts. This required that $abs(L_1 - L_2) \geq 1$, where L_1 = log fold change for H_2O_2 vs. mock and L_2 = log fold change for La^{3+} + H_2O_2 treatment vs. La^{3+} only. For each of the protein groups that passed the filters, we extracted all identifiers in the group. For identifiers that occurred in multiple groups, we removed the identifier from the group where it occurred the least.

CKN network analysis

For each Ca^{2+}-dependent redox-responsive protein group (target), we identified the closest nodes upstream that had a known Ca^{2+}-signaling association (source). This was done by identifying all shortest paths in CKN with the source nodes set as all genes with Ca^{2+}-signaling-related GoMapMan (Ramšak et al., 2014) annotations and the target set as the

Ca^{2+}-dependent H_2O_2-responsive peptides. The GoMapMan annotations considered were "30.3 - signaling.calcium," "34.21 - transport.calcium," and "34.22 - transport.cyclic nucleotide or calcium regulated channels." For each target, we kept the source(s) with the shortest paths to the target (the "closest" upstream potential Ca^{2+} interactor). We used the CUT-tool on the merged network to determine the cut-set between all the source nodes and each target. The capacity on the edges was set as the edge rank + 1 (highly ranked edges are more likely to be in the cut-set).

All source and target nodes are listed in Supplemental Table 4, and the complete network is available to view as a high-quality pdf in Supplemental Figure 3. Analysis was performed in Python using the networkX library (Hagberg et al., 2008) and visualized in Cytoscape (Cline et al., 2007) using the py4cytoscape library (Ono et al., 2021). All code is available in the SKM-tools repository (https://github.com/NIB-SI/skm-tools).

Gene identifiers

All genes mentioned in the article are listed with their gene identifiers in Supplemental Table 10.

SUPPLEMENTAL INFORMATION

Supplemental information is available at *Plant Communications Online*.

FUNDING

SKM was developed with funding from the European Union's Horizon 2020 research and innovation programme under grant agreement 862858 (ADAPT); the Slovenian Research Agency under grant agreements 1000-15-0105, Z7-1888, J4-1777, P4-0165, N4-0199, Z4-50146, and J4-3089; and ELIXIR, the research infrastructure for life science data through the ELIXIR Implementation Study "Increasing plant data findability for ELIXIR and beyond" and ELIXIR-SI. We gratefully acknowledge funding from the Deutsche Forschungsgemeinschaft (DFG) to U.C.V. (INST 217/939-1 FUGG).

AUTHOR CONTRIBUTIONS

For SKM: software and visualization, C.B. and V.P.; data curation of CKN, Ž.R. and C.B.; data curation of PSS, C.B., Ž.R., M.Z., Š.B., M.P., M.K., A.Ž., and K.G.; supervision, project administration, and funding acquisition, KG. For case studies: methodology, L.A.-S. and W.W.; investigation, A.B., B.W., A.v.D., J.G., and L.A.-S.; formal analysis, A.B. and C.B.; data curation, A.B., B.W., A.v.D., L.A.-S., M.Z., and Š.B.; visualization, C.B., A.B., and M.Z.; supervision, U.C.V., M.T., and K.G.; project administration and funding acquisition, U.C.V., M.T., and K.G. Writing – original draft, C.B., A.B., Ž.R., and K.G. All authors took part in writing – review & editing.

ACKNOWLEDGMENTS

We would like to acknowledge Solana Research GmbH for producing *StRD29::fluc* Désirée transgenic potatoes and Nelly Braun for preselection of the *StRD29* lines. We would also like to thank the many additional contributors to PSS: Anna Coll, Barbara Jaklič, Christian Bachem, Christian Schuy, Juan Antonio López-Ráez, Katja Stare, Maria Pozo, Mojca Juteršek, Špela Tomaž, Tim Godec, Tjaša Lukan, Tjaša Mahkovec Povalej, Valentina Levak, Vaňková Radka, Vid Modic, and Maroof Shaikh. For support in integration of SKM into FAIDARE, we would like to thank Cyril Pommier. Part of this work was performed during the 2nd BioHackathon Germany in Bielefeld, organized by de.NBI and ELIXIR Germany in December 2023. Finally, we would like to acknowledge Zoran Nikoloski for discussions regarding the CKN analyses. No conflict of interest is declared.

Received: December 28, 2023
Revised: March 28, 2024
Accepted: April 11, 2024
Published: April 15, 2024

REFERENCES

Aleman, F., Yazaki, J., Lee, M., Takahashi, Y., Kim, A.Y., Li, Z., Kinoshita, T., Ecker, J.R., and Schroeder, J.I. (2016). An ABA-increased interaction of the PYL6 ABA receptor with MYC2 Transcription Factor: A putative link of ABA and JA signaling. Sci. Rep. **6**, 28941.

Aric, H., Schult, D., and Renieris, M. (2024). PyGraphviz [Computer software]. https://pygraphviz.github.io.

Arvidsson, S., Kwasniewski, M., Riaño-Pachón, D.M., and Mueller-Roeber, B. (2008). QuantPrime – a flexible tool for reliable high-throughput primer design for quantitative PCR. BMC Bioinf. **9**:465.

Baebler, Š., Svalina, M., Petek, M., Stare, K., Rotter, A., Pompe-Novak, M., and Gruden, K. (2017). quantGenius: implementation of a decision support system for qPCR-based gene quantification. BMC Bioinf. **18**:276.

Baker, S.S., Wilhelm, K.S., and Thomashow, M.F. (1994). The 5′-region of Arabidopsis thaliana cor15a has cis-acting elements that confer cold-drought- and ABA-regulated gene expression. Plant Mol. Biol. **24**:701–713.

Balci, H., Siper, M.C., Saleh, N., Safarli, I., Roy, L., Kilicarslan, M., Ozaydin, R., Mazein, A., Auffray, C., Babur, Ö., et al. (2021). Newt: a comprehensive web-based tool for viewing, constructing and analyzing biological maps. Bioinformatics **37**:1475–1477.

Berardini, T.Z., Reiser, L., Li, D., Mezheritsky, Y., Muller, R., Strait, E., and Huala, E. (2015). The arabidopsis information resource: Making and mining the "gold standard" annotated reference plant genome. genesis **53**:474–485.

Bergmann, F.T., Czauderna, T., Dogrusoz, U., Rougny, A., Dräger, A., Touré, V., Mazein, A., Blinov, M.L., and Luna, A. (2020). Systems biology graphical notation markup language (SBGNML) version 0.3. J. Integr. Bioinforma **17**.

Bittner, A., Cieśla, A., Gruden, K., Lukan, T., Mahmud, S., Teige, M., Vothknecht, U.C., and Wurzinger, B. (2022). Organelles and phytohormones: a network of interactions in plant stress responses. J. Exp. Bot. **73**:7165–7181.

Bornstein, B.J., Keating, S.M., Jouraku, A., and Hucka, M. (2008). LibSBML: an API Library for SBML. Bioinformatics **24**:880–881.

Caspi, R., Billington, R., Ferrer, L., Foerster, H., Fulcher, C.A., Keseler, I.M., Kothari, A., Krummenacker, M., Latendresse, M., Mueller, L.A., et al. (2016). The MetaCyc database of metabolic pathways and enzymes and the BioCyc collection of pathway/genome databases. Nucleic Acids Res. **44**:D471–D480.

Cheng, C.-Y., Krishnakumar, V., Chan, A.P., Thibaud-Nissen, F., Schobel, S., and Town, C.D. (2017). Araport11: a complete reannotation of the Arabidopsis thaliana reference genome. Plant J. **89**:789–804.

Chow, C.-N., Lee, T.-Y., Hung, Y.-C., Li, G.-Z., Tseng, K.-C., Liu, Y.-H., Kuo, P.-L., Zheng, H.-Q., and Chang, W.-C. (2019). PlantPAN3.0: a new and updated resource for reconstructing transcriptional regulatory networks from ChIP-seq experiments in plants. Nucleic Acids Res. **47**:D1155–D1163.

Cline, M.S., Smoot, M., Cerami, E., Kuchinsky, A., Landys, N., Workman, C., Christmas, R., Avila-Campilo, I., Creech, M., Gross, B., et al. (2007). Integration of biological networks and gene expression data using Cytoscape. Nat. Protoc. **2**:2366–2382.

Cusack, S.A., Wang, P., Lotreck, S.G., Moore, B.M., Meng, F., Conner, J.K., Krysan, P.J., Lehti-Shiu, M.D., and Shiu, S.-H. (2021). Predictive Models of Genetic Redundancy in Arabidopsis thaliana. Mol. Biol. Evol. **38**:3397–3414.

Eckardt, N.A. (2015). The Plant Cell Reviews Dynamic Aspects of Plant Hormone Signaling and Crosstalk. Plant Cell **27**:1–2.

Edmonds, J., and Karp, R.M. (1972). Theoretical Improvements in Algorithmic Efficiency for Network Flow Problems. J. ACM **19**:248–264.

Foix, L., Nadal, A., Zagorščak, M., Ramšak, Ž., Esteve-Codina, A., Gruden, K., and Pla, M. (2021). Prunus persica plant endogenous peptides PpPep1 and PpPep2 cause PTI-like transcriptome reprogramming in peach and enhance resistance to Xanthomonas arboricola pv. pruni. BMC Genom. **22**:360.

Gansner, E.R., and North, S.C. (2000). An open graph visualization system and its applications to software engineering. Software Pract. Ex. **30**:1203–1233.

Garrett, K.A. (2013). Agricultural impacts: Big data insights into pest spread. Nat. Clim. Change **3**:955–957.

Hagberg, A.A., Schult, D.A., and Swart, P.J. (2008). Exploring Network Structure, Dynamics, and Function using NetworkX. In Proceedings of the 7th Python in Science Conference (SciPy 2008) (Pasadena).

Hassani-Pak, K., Singh, A., Brandizi, M., Hearnshaw, J., Parsons, J.D., Amberkar, S., Phillips, A.L., Doonan, J.H., and Rawlings, C. (2021). KnetMiner: a comprehensive approach for supporting evidence-based gene discovery and complex trait analysis across species. Plant Biotechnol. J. **19**:1670–1678.

Hastings, J., Owen, G., Dekker, A., Ennis, M., Kale, N., Muthukrishnan, V., Turner, S., Swainston, N., Mendes, P., and Steinbeck, C. (2016). ChEBI in 2016: Improved services and an expanding collection of metabolites. Nucleic Acids Res. **44**:D1214–D1219.

Herwig, R., Hardt, C., Lienhard, M., and Kamburov, A. (2016). Analyzing and interpreting genome data at the network level with ConsensusPathDB. Nat. Protoc. **11**:1889–1907.

Hunter, M.C., Smith, R.G., Schipanski, M.E., Atwood, L.W., and Mortensen, D.A. (2017). Agriculture in 2050: Recalibrating targets for sustainable intensification. Bioscience **67**:386–391.

IPPC Secretariat. (2021). Scientific Review of the Impact of Climate Change on Plant Pests – A Global Challenge to Prevent and Mitigate Plant Pest Risks in Agriculture, Forestry and Ecosystems (Rome: FAO on behalf of the IPPC Secretariat).

Juteršek, M., Petek, M., Ramšak, Ž., Moreno-Giménez, E., Gianoglio, S., Mateos-Fernández, R., Orzáez, D., Gruden, K., and Baebler, Š. (2022). Transcriptional deregulation of stress-growth balance in Nicotiana benthamiana biofactories producing insect sex pheromones. Front. Plant Sci. **13**, 941338.

Kanehisa, M., Sato, Y., Kawashima, M., Furumichi, M., and Tanabe, M. (2016). KEGG as a reference resource for gene and protein annotation. Nucleic Acids Res. **44**:D457–D462.

Keating, S.M., Waltemath, D., König, M., Zhang, F., Dräger, A., Chaouiya, C., Bergmann, F.T., Finney, A., Gillespie, C.S., Helikar, T., et al. (2020). SBML Level 3: an extensible format for the exchange and reuse of biological models. Mol. Syst. Biol. **16**, e9110.

Ono, K., Bouças, J., Nishida, K., and Demchak, B. (2021). py4cytoscape [Computer software]. https://py4cytoscape.readthedocs.io.

Klarner, H., Streck, A., and Siebert, H. (2017). PyBoolNet: A python package for the generation, analysis and visualization of boolean networks. Bioinformatics **33**:770–772.

Knight, M.R., Smith, S.M., and Trewavas, A.J. (1992). Wind-induced plant motion immediately increases cytosolic calcium. Proc. Natl. Acad. Sci. USA **89**:4967–4971.

König, M. (2020). matthiaskoenig/libsbgn-python: 0.2.2 Advance Access. https://doi.org/10.5281/zenodo.4171366.

Kudla, J., Batistič, O., and Hashimoto, K. (2010). Calcium Signals: The Lead Currency of Plant Information Processing. Plant Cell **22**:541–563.

Lichtenberg, J., Yilmaz, A., Welch, J.D., Kurz, K., Liang, X., Drews, F., Ecker, K., Lee, S.S., Geisler, M., Grotewold, E., and Welch, L.R. (2009). The word landscape of the non-coding segments of the Arabidopsis thaliana genome. BMC Genom. **10**:463.

Miljkovic, D., Stare, T., Mozetič, I., Podpečan, V., Petek, M., Witek, K., Dermastia, M., Lavrač, N., and Gruden, K. (2012). Signalling Network Construction for Modelling Plant Defence Response. PLoS One **7**, e51822.

Mueller, L.A., Zhang, P., and Rhee, S.Y. (2003). AraCyc: A Biochemical Pathway Database for Arabidopsis. Plant Physiol. **132**:453–460.

Mur, L.A.J., Kenton, P., Atzorn, R., Miersch, O., and Wasternack, C. (2006). The Outcomes of Concentration-Specific Interactions between Salicylate and Jasmonate Signaling Include Synergy, Antagonism, and Oxidative Stress Leading to Cell Death. Plant Physiol. **140**:249–262.

Müssel, C., Hopfensitz, M., and Kestler, H.A. (2010). BoolNet – an R package for generation, reconstruction and analysis of Boolean networks. Bioinformatics **26**:1378–1380.

Nomoto, M., Skelly, M.J., Itaya, T., Mori, T., Suzuki, T., Matsushita, T., Tokizawa, M., Kuwata, K., Mori, H., Yamamoto, Y.Y., et al. (2021). Suppression of MYC transcription activators by the immune cofactor NPR1 fine-tunes plant immune responses. Cell Rep. **37**, 110125.

Otasek, D., Morris, J.H., Bouças, J., Pico, A.R., and Demchak, B. (2019). Cytoscape Automation: empowering workflow-based network analysis. Genome Biol. **20**:185.

Peixoto, T.P. (2014). The graph-tool python library (*figshare* Advance). https://doi.org/10.6084/m9.figshare.1164194.

Pirayesh, N., Giridhar, M., Ben Khedher, A., Vothknecht, U.C., and Chigri, F. (2021). Organellar calcium signaling in plants: An update. Biochim. Biophys. Acta Mol. Cell Res. **1868**, 118948.

Podpečan, V. (2023). Vpodpecan/Pysbgn: v0.2.1 Advance Access. https://doi.org/10.5281/zenodo.7966410.

Podpečan, V., Ramšak, Ž., Gruden, K., Toivonen, H., and Lavrač, N. (2019). Interactive exploration of heterogeneous biological networks with Biomine Explorer. Bioinformatics **35**:5385–5388.

Pylianidis, C., Osinga, S., and Athanasiadis, I.N. (2021). Introducing digital twins to agriculture. Comput. Electron. Agric. **184**, 105942.

Ramsak, Ž., Baebler, Š., Rotter, A., Korbar, M., Mozetič, I., Usadel, B., and Gruden, K. (2014). GoMapMan: integration, consolidation and visualization of plant gene annotations within the MapMan ontology. Nucleic Acids Res. **42**:D1167–D1175.

Ramšak, Ž., Coll, A., Stare, T., Tzfadia, O., Baebler, Š., Van de Peer, Y., and Gruden, K. (2018). Network modeling unravels mechanisms of crosstalk between ethylene and salicylate signaling in potato. Plant Physiol. **178**:488–499.

Rentel, M.C., and Knight, M.R. (2004). Oxidative Stress-Induced Calcium Signaling in Arabidopsis. Plant Physiol. **135**:1471–1479.

Rocha-Sosa, M., Sonnewald, U., Frommer, W., Stratmann, M., Schell, J., and Willmitzer, L. (1989). Both developmental and metabolic signals activate the promoter of a class I patatin gene. EMBO J. **8**:23–29.

Sebastian, K., Nowee, P., and Carrera, E. (2023). Pydot Advance Access.

Shannon, P., Markiel, A., Ozier, O., Baliga, N.S., Wang, J.T., Ramage, D., Amin, N., Schwikowski, B., and Ideker, T. (2003). Cytoscape: A Software Environment for Integrated Models of Biomolecular Interaction Networks. Genome Res. **13**:2498–2504.

(2022). IPCC, 2022: Summary for Policymakers. In Climate Change 2022: Mitigation of Climate Change. Contribution of Working Group III to the Sixth Assessment Report of the Intergovernmental Panel on Climate Change, P.R. Shukla, J. Skea, R. Slade, A. Al Khourdajie, R. van Diemen, D. McCollum, M. Pathak, S. Some, R. Vyas, and M. Fradera, et al., eds. (Cambridge University Press).

Steinwand, M.A., and Ronald, P.C. (2020). Crop biotechnology and the future of food. Nat. Food **1**:273–283.

Stuhlfelder, C., Lottspeich, F., and Mueller, M.J. (2002). Purification and partial amino acid sequences of an esterase from tomato. Phytochemistry **60**:233–240.

Szklarczyk, D., Kirsch, R., Koutrouli, M., Nastou, K., Mehryary, F., Hachilif, R., Gable, A.L., Fang, T., Doncheva, N.T., Pyysalo, S., et al. (2023). The STRING database in 2023: protein–protein association networks and functional enrichment analyses for any sequenced genome of interest. Nucleic Acids Res. **51**:D638–D646.

Tracy, F.E., Gilliham, M., Dodd, A.N., Webb, A.a.R., and Tester, M. (2008). NaCl-induced changes in cytosolic free Ca2+ in Arabidopsis thaliana are heterogeneous and modified by external ionic composition. Plant Cell Environ. **31**:1063–1073.

Tyanova, S., Temu, T., Sinitcyn, P., Carlson, A., Hein, M.Y., Geiger, T., Mann, M., and Cox, J. (2016a). The Perseus computational platform for comprehensive analysis of (prote)omics data. Nat. Methods **13**:731–740.

Tyanova, S., Temu, T., and Cox, J. (2016b). The MaxQuant computational platform for mass spectrometry-based shotgun proteomics. Nat. Protoc. **11**:2301–2319.

United Nations Department of Economic and Social Affairs, Population Division. (2022). World Population Prospects 2022: Summary of Results.

Van Bel, M., Silvestri, F., Weitz, E.M., Kreft, L., Botzki, A., Coppens, F., and Vandepoele, K. (2022). PLAZA 5.0: extending the scope and power of comparative and functional genomics in plants. Nucleic Acids Res. **50**:D1468–D1474.

Wilkinson, M.D., Dumontier, M., Aalbersberg, I.J., Appleton, G., Axton, M., Baak, A., Blomberg, N., Boiten, J.-W., da Silva Santos, L.B., Bourne, P.E., et al. (2016). The FAIR Guiding Principles for scientific data management and stewardship. Sci. Data **3**, 160018.

Wu, J., Wang, L., and Baldwin, I.T. (2008). Methyl jasmonate-elicited herbivore resistance: does MeJA function as a signal without being hydrolyzed to JA? Planta **227**:1161–1168.

Zagorščak, M., Blejec, A., Ramšak, Ž., Petek, M., Stare, T., and Gruden, K. (2018). DiNAR: revealing hidden patterns of plant signalling dynamics using Differential Network Analysis in R. Plant Methods **14**:78.

Zhang, N., Zhou, S., Yang, D., and Fan, Z. (2020). Revealing Shared and Distinct Genes Responding to JA and SA Signaling in Arabidopsis by Meta-Analysis. Front. Plant Sci. **11**.

Plant Communications, Volume *5*

Supplemental information

Stress Knowledge Map: A knowledge graph resource for systems biology analysis of plant stress responses

Carissa Bleker, Živa Ramšak, Andras Bittner, Vid Podpečan, Maja Zagorščak, Bernhard Wurzinger, Špela Baebler, Marko Petek, Maja Križnik, Annelotte van Dieren, Juliane Gruber, Leila Afjehi-Sadat, Wolfram Weckwerth, Anže Županič, Markus Teige, Ute C. Vothknecht, and Kristina Gruden

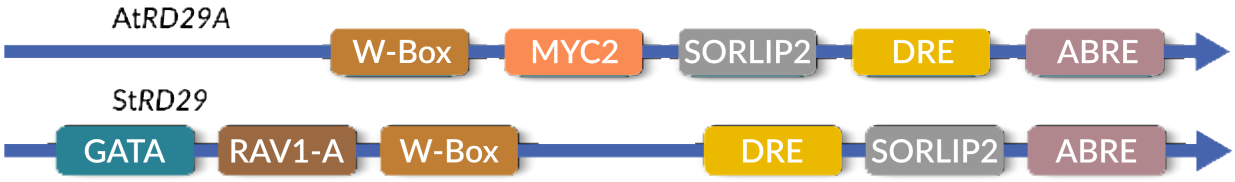

Supplementary Figure 1: Visualisation of abiotic stress related cis-regulatory binding motifs within the 1 kbp upstream region of the transcription initiation site of AtRD29A and StRD29.
ABRE: ABA-Responsive Element; DRE: Dehydration Responsive Elementi; GATA-Box: light responsive GATA motif; SORLIP2: Sequence Over-Represented in Light-Induced Promoters; RAV1-A: RAV1 binding sequence; WRKY: W-Box recognition element; MYC2: basic-helix-loop-helix transcription factor MYC2 binding site.

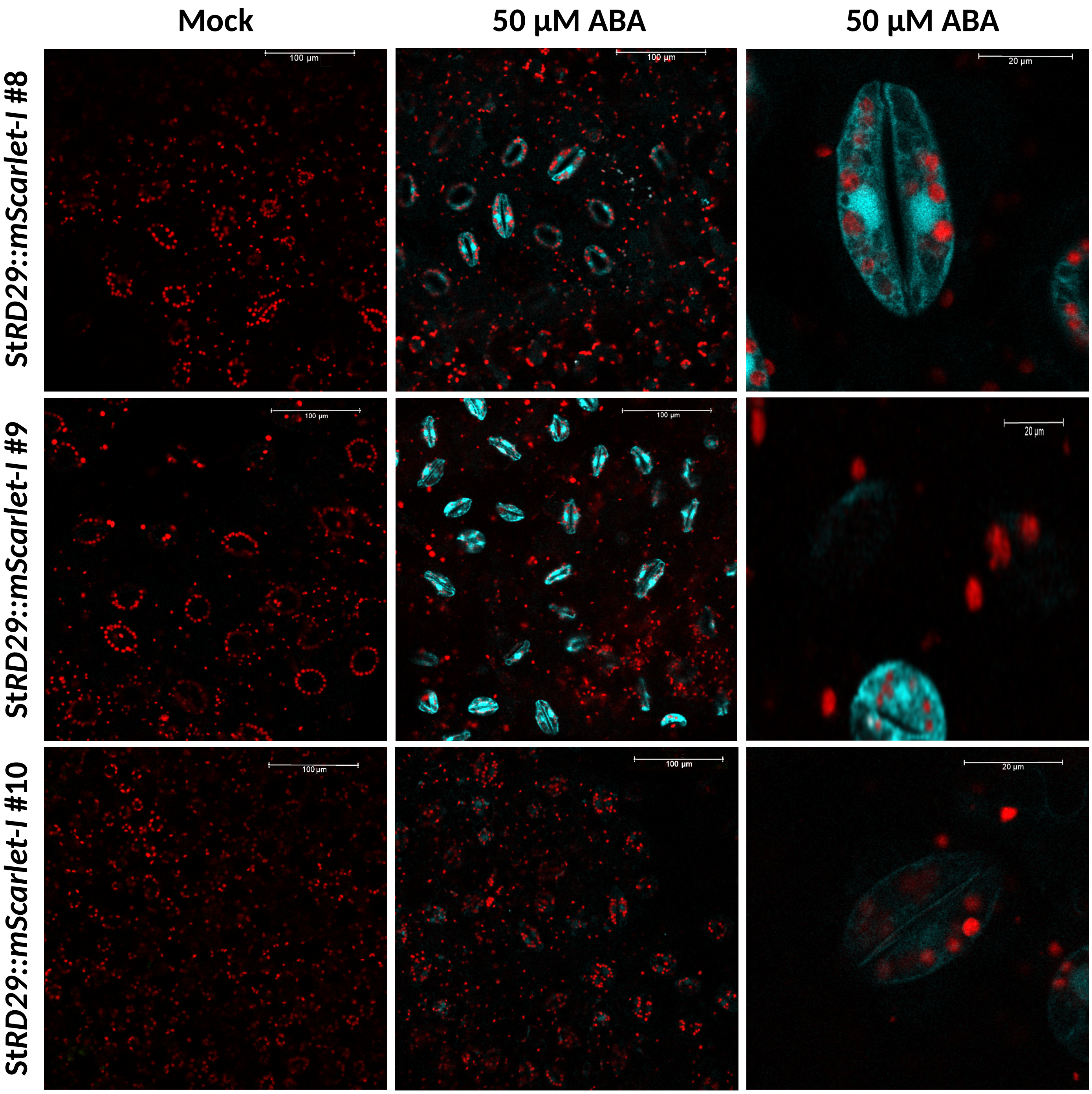

Supplementary Figure 2: Microscopic analyses of the ABA response of *StRD29::mScarlet-I.*
The fluorophore mScarlet-I was expressed under the control of the StRD29 promoter in transgenic potato plants. Leaf discs (7 mm) of three differentlines (#10 with very weak response) were treated with 50 µM ABA (or imaging buffer as a mock control) for 24 hours. After the incubation, mScarlet-I fluorescence was visualized with an excitation at 569 nm and emission was recorded at 585-595 nm (cyan) using a Leica SP8 lightning. Chlorophyll fluorescence (red) was visualized with an excitation at 569 nm with emission recorded at 650 – 705 nm.

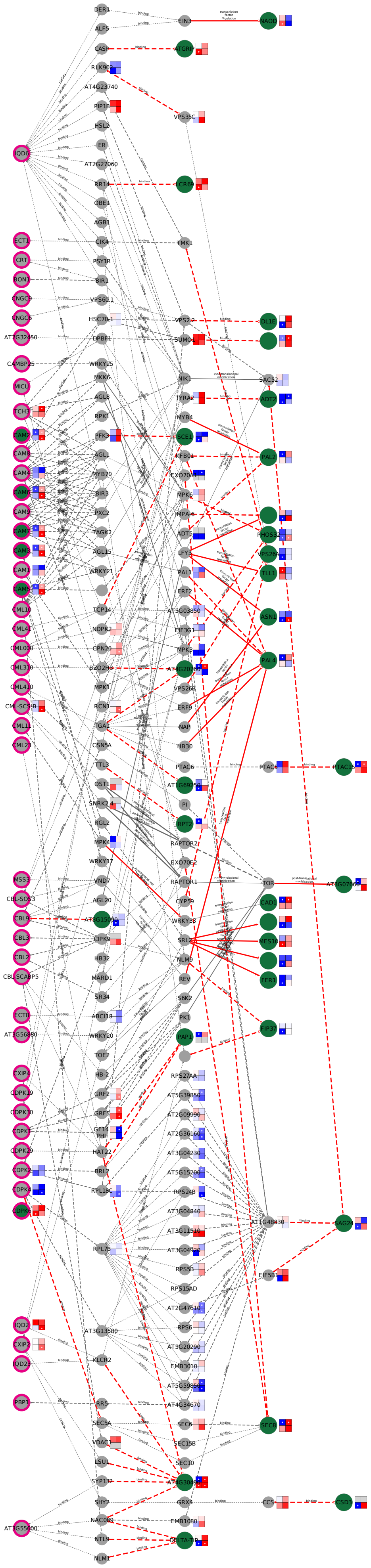

Supplementary Figure 3: Deciphering the Ca^{2+} dependent network in peroxide signalling.
All shortest paths identified in CKN leading from known Ca^{2+} related proteins (sources - pink bordered nodes) to Ca^{2+}-dependent redox-responsive proteins identified by proteomics (targets - green filled nodes) using rank 0, rank 1, and rank 2 edges (as described in Table 1 legend), merged into a single network. Legend as in Figure 4.

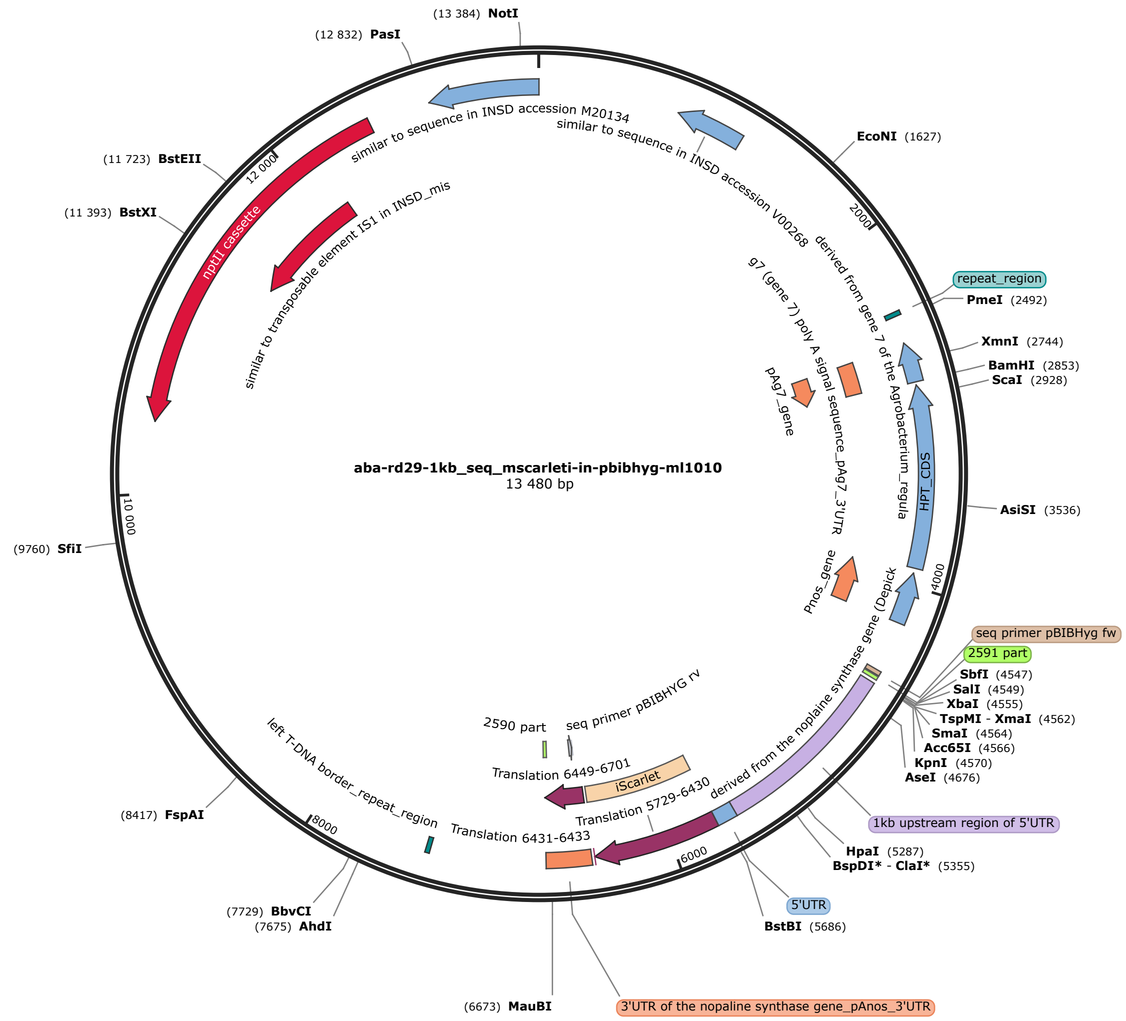

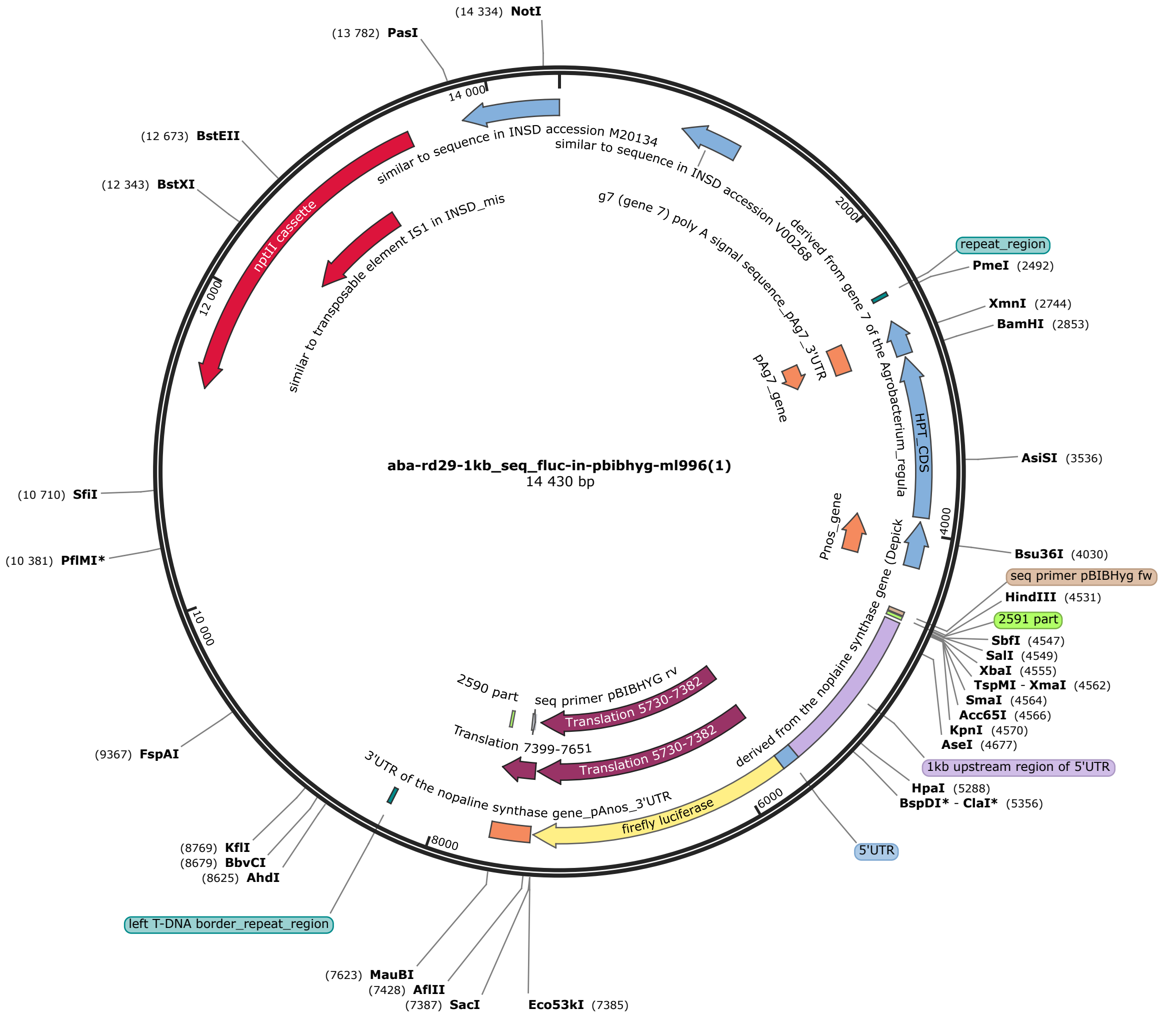

Supplementary Figure 4: Visualisation of the features of Vector pBibHyg carrying StRD29::mScarletI. (above) and StRD29::fluc (below).
